# The Role of Functional Groups in Substituted Benzoic Acids Used as Dopants in Liquid Crystal Mixtures on the Nematic–Isotropic Transitions

**DOI:** 10.1021/acs.jpcb.6c01531

**Published:** 2026-07-01

**Authors:** María Celina Mora, Joshua Brenes, Cristopher Camacho, Erick Castellón

**Affiliations:** † Escuela de Química, 27915 Universidad de Costa Rica, San José 11501-2060, Costa Rica; ‡ Centro de Investigación en Ciencia y Tecnología de Materiales, Universidad de Costa Rica, San José 11501-2060, Costa Rica

## Abstract

Thermotropic nematic liquid crystals (LC) are orientationally ordered liquids composed of mesogenic molecules that become isotropic liquids at the nematic–isotropic transition temperature (*T*_NI_), at which the order disappears. Dissolving a nonmesogenic substance in an LC usually produces a disruption of the molecular order that leads to a decreasing of the *T*_NI_. However, some nonmesogenic substances enhance the molecular order, yielding increments of the *T*_NI_. Rigid aromatic carboxylic acids like benzoic acid possess this order-enhancing capacity due to the formation of elongated dimers, which align with the uniaxial nematic environment. In the present research, the thermodynamics of the dimerization of 20 substituted benzoic acids dissolved in the nematic LC 4-*n*-pentyl-4′-cyanobiphenyl (5CB) and the phase behavior of the mixtures were assessed. The substituents include amino, fluoro, chloro, bromo, iodo, and nitro groups linked to the aromatic ring of the benzoic acid at *ortho*, *meta*, and *para* positions. Both the geometry and the electron-withdrawing or donating effect of the substituent have an influence on the ordering capability of the substituted benzoic acid. In general, electron-donating groups at the *para* position in benzoic acids have a large order-enhancing power. Electron-withdrawing groups promote the ionization of the substituted benzoic acids, limiting the dimerization capability of these dopants. Regarding the substitution position of the groups, the *para* substitutions lead to the formation of long and slim calamitic dimers with a large aspect ratio length/diameter. These calamitic dimers align with the nematic solvent and act as scaffolds in the uniaxial solvent. The nematic LC mixtures were analyzed by polarized optical microscopy, differential scanning calorimetry, and infrared spectroscopy. Theoretical calculations aided to ascertain the most probable dimers in amphoteric dopants like aminobenzoic acids. A thermodynamic model was developed to analyze the nematic–isotropic transitions. Two dopants, 4-aminobenzoic acid and 4-*N*-methylaminobenzoic acid, exhibit extraordinary order-enhancing power, yielding increments of the nematic–isotropic transition temperature up to 12 K with minor concentration of the dopants.

## Graphical abstract

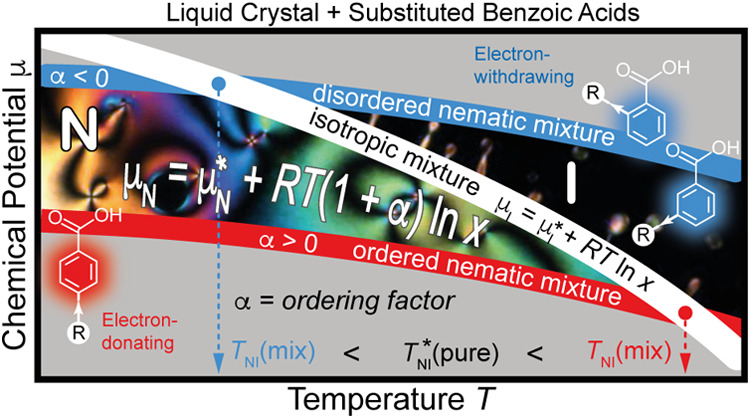

## Introduction

Liquid crystals (LCs) are mesophases (intermediate phases) between crystalline solids and isotropic liquids.^1,2^ These mesophases exhibit anisotropic properties, arising from their molecular structure and ordering. This state of matter combines the molecular order typical of crystalline solids with the fluidity of liquids, thereby imparting optical and electrical anisotropy.^3,4^ Such characteristics are exploited in a variety of electro-optical devices, including displays, thermometers, and smart windows.^5,6^ The most widely known application of LCs is in liquid-crystal displays, where their optical properties are controlled by the application of an external electric field that interacts with the molecular dipole moment, inducing the reorientation of the molecules.^7,8^

Mesogens are substances that generate mesophases. For a compound to exhibit a thermotropic liquid-crystalline mesophase, its molecular structure should combine a rigid core, typically composed of aromatic rings, and flexible side chains such as alkyl or alkoxy groups.^1,2,9^ This molecular configuration promotes the coexistence of order and mobility in the mesophase.^9^

Nematic thermotropic liquid crystals undergo a phase transition to an isotropic liquid state at a specific temperature known as the nematic–isotropic transition temperature (*T*_NI_). At this point, the orientational order characteristic of the nematic mesophase is lost.

The nematic–isotropic transition temperature *T*_NI_ can be altered by the presence of doping dispersed particles or dissolved molecules in a liquid crystal. Mixing liquid-crystalline mesogens with other mesogens can generate LC eutectic mixtures with a broad temperature interval of mesophase behavior.^10^ On the contrary, when a nematic liquid crystal is mixed with a nonmesogenic substance, frequently this dopant substance disrupts to some extent the molecular order of the liquid crystal, decreasing the isotropization temperature.^11^ However, there are some instances where blending a liquid crystal with nonmesogenic substances or nanoparticles raises *T*_NI_. Doping the LC mixture E7 with carbon nanotubes (0.1–0.2% in weight) leads to an increase of *T*_NI_ due to the anisotropic alignment induced by the nanotubes on the liquid crystal molecules.^12^ Another example is the isotropization temperature rise of 9 °C achieved by blending ferroelectric nanoparticles of BaTiO_3_ with the LC MLC-6609 at low concentrations of particles (0.2%).^13,14^ In this case, the spatial distribution of dipole moments of the nanoparticles couples with the orientational order of the LC, increasing the molecular ordering of the mixture.^15^ Regarding nonmesogenic molecular dopants, the addition of 4-aminobiphenyl to the LC 4-*n*-pentyl-4′-cyanobiphenyl (5CB) increases *T*_NI_ up to 4 °C.^16^ Recently, salicylaldoxime and resorcinol have been reported to increase the *T*_NI_ of their mixtures with 5CB due to the formation of hydrogen-bonded complexes with the mesogen, with an increase of the nematic–isotropic transition of 16 °C for a mixture 5CB + resorcinol with a molar proportion of 3:1.^17,18^ Also, nonmesogenic aromatic carboxylic acids (like benzoic acid or biphenyl-4-carboxylic acid) added to 5CB LC have an order-enhancing effect explained by the formation of long and rigid dimers of the acids in the nematic solvent, causing an increasing of the nematic–isotropic transition temperature.^19^

The present research deepens on the study of benzoic acids used as dopants in mixtures with the nematic liquid crystal 4-*n*-pentyl-4′-cyanobiphenyl (5CB). Twenty (20) benzoic acids functionalized with halogen, nitro, and amino groups at varied positions in the aromatic ring are added as dopants for the 5CB. The nematic–isotropic transitions of the mixtures are assessed. The interplay between molecular geometry and electron-withdrawing capabilities of the substituent groups in the functionalized benzoic acids is discussed. The molecular association of the functionalized benzoic acids to form dimers is evaluated by infrared spectroscopy, and a thermodynamic model is developed to analyze the experimental data. Some of the assessed dopants showed a colossal order-enhancing effect at low concentrations, becoming promising candidates for their use in liquid crystal mixtures with an expanded nematic range of temperature.

## Experimental Section

### Materials

The nematic liquid crystal 4-*n*-pentyl-4′-cyanobiphenyl (5CB, ABCR, Germany) was used in the experiments, which in its pure state exhibits a nematic–isotropic transition temperature (*T*_NI_) at 35.5 °C. The 5CB was mixed with 2-aminobenzoic acid, 3-aminobenzoic acid, 4-aminobenzoic acid, 4-*N*-methylaminobenzoic acid, 2-bromobenzoic acid, 3-bromobenzoic acid, 4-bromobenzoic acid, 2-fluorobenzoic acid, 3-fluorobenzoic acid, 4-fluorobenzoic acid, 2-iodobenzoic acid, 3-iodobenzoic acid, 4-iodobenzoic acid, 2-chlorobenzoic acid, 3-chlorobenzoic acid, 4-chlorobenzoic acid, 2-nitrobenzoic acid, 3-nitrobenzoic acid, 3,5-dinitrobenzoic acid, and 4-nitrobenzoic acid (Sigma-Aldrich, USA). The chemical structures and tag numbers of these dopants are displayed in Figure 1. Other reagents used as solvents for the acids, for cleaning and functionalizing substrates, were ethanol, tetrahydrofuran (THF), and poly­(vinyl alcohol) (PVA), purchased from Sigma-Aldrich and used as received.

**Figure 1:**
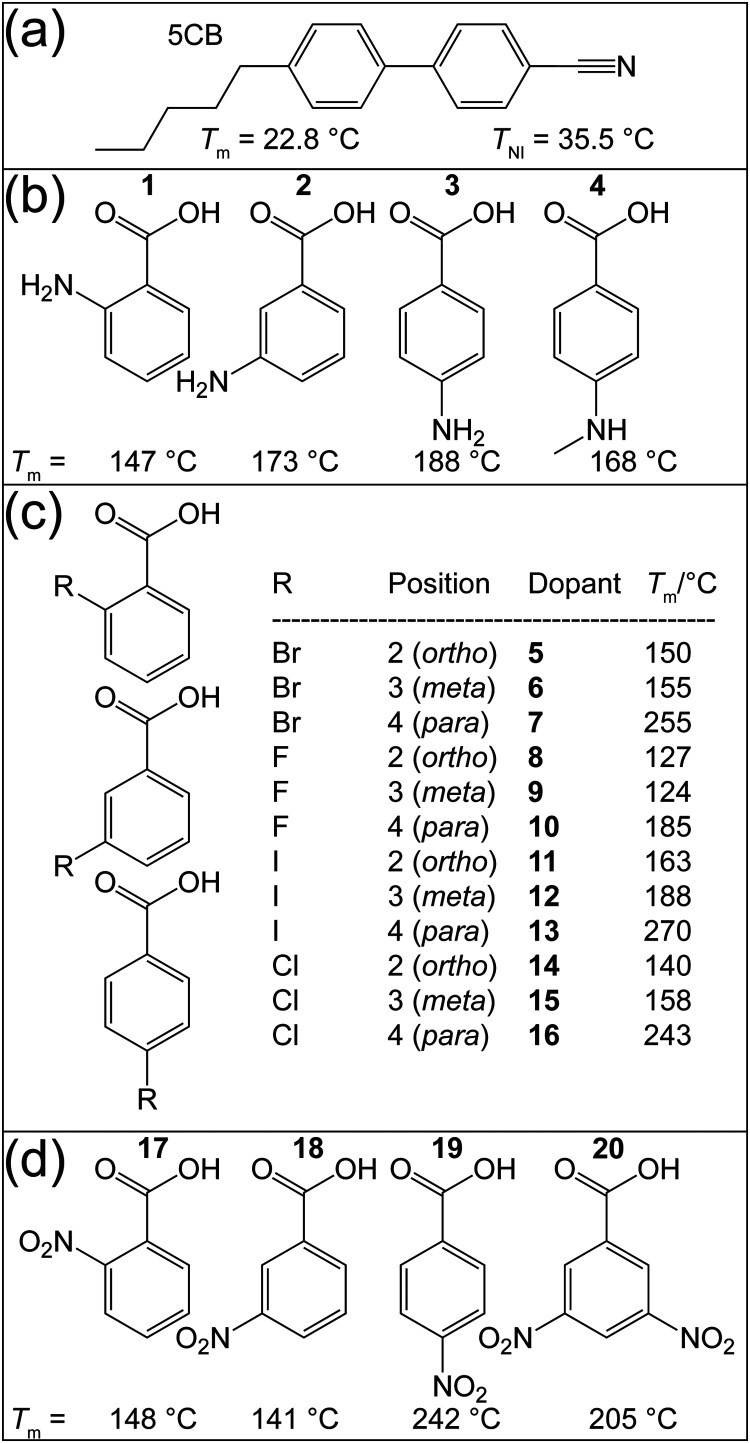
Chemical structures of the liquid crystal and dopants. (a) Nematic liquid crystal 5CB. (b) Aminobenzoic acids. (c) Halobenzoic acids. (d) Nitrobenzoic acids. The reported melting temperatures (*T*_*m*_) of 5CB LC^20^ and substituted benzoic acids^21^ are displayed.

### LC Mixtures

Solutions of the acids were prepared at concentrations ranging from 20 to 50 g/L in ethanol or THF, depending on the reported solubility of each acid in these solvents. Measured volumes (30–500 μL) of these solutions were mixed with 5CB (150.00 mg, exactly measured) in vials to achieve homogeneous mixtures at three different acid concentrations (nominal molar fractions *x*_A_^′^ = 0.005–0.06). Each mixture vial (volatile solvent + 5CB + carboxylic acid) was heated in an oven under reduced pressure at temperatures between 70 and 80 °C for 24 h to ensure complete solvent evaporation and to create acid-doped liquid crystal mixtures.

### Polarized Optical Microscopy

The nematic–isotropic transitions of the mixtures were observed using a polarized optical microscope (Olympus BX53 Pol) equipped with a digital camera (FLIR Grasshopper 3) connected to a computer. Sample temperatures were controlled using a heating stage (Linkam) with a resolution of 0.1 °C. All samples were observed with a magnification of 10× between crossed polarizers set at 90° to characterize the mesophases. Each sample was placed between two microscope glasses previously coated with a thin layer of poly­(vinyl alcohol) (PVA) to ensure planar degenerate anchoring (i.e., the molecular orientation lies within the plane, without a preferred in-plane direction). The PVA coatings were prepared by spreading 10 μL of an aqueous solution of the polymer (1% PVA) onto the glass substrates and drying them at 100 °C to consolidate the thin PVA film.

To induce phase transitions in the LC mixtures, each sample under observation in the microscope was subjected to a temperature program consisting of the following steps: heating at rate 15 °C/min from 25 °C up to a temperature 2 °C below the isotropization temperature (*T*_NI_), near to the *T*_NI_, the heating rate was 1 °C/min until the isotropization of the sample (characterized by a dark observation field). The top temperature was set to 2 °C above *T*_NI_. The next step was cooling at rate −1 °C/min up to a temperature 2 °C below *T*_NI_, followed by cooling at rate −15 °C/min to reach the final temperature of 25 °C. The steps with slow increasing or decreasing temperatures were set to carefully observe the phase transitions. Micrographs were taken every 15 s during the rapid heating and cooling ramps, and every 2 s near the nematic–isotropic transition temperature of the LC mixtures.

### Differential Scanning Calorimetry

The isotropization enthalpy of the mixtures was measured using a differential scanning calorimeter (Mettler Toledo DSC 3+). Two sealed aluminum crucibles were used: one containing the liquid crystal mixture (∼5 mg) and the other left empty as the reference crucible. To induce the nematic–isotropic transition, the temperature of the samples was increased from 25 to 60 °C at rate 10 °C/min, followed by cooling at rate −10 °C/min, finishing the temperature cycle at 25 °C. The selected heating and cooling rates for the DSC experiments produced well-defined heat flow peaks, appropriate for measuring the transition temperatures and associated enthalpies. The transition temperatures (*T*_NI_) were determined by identifying the peak maxima and minima in the heat flow curves (thermograms). Numerical details for determining transition enthalpies are provided in the Supporting Information.

### Infrared Spectra

The intermolecular interactions of the LC mixtures were analyzed with a Fourier transform infrared (FTIR) spectrometer (PerkinElmer Frontier). Samples of 5CB + acid (nominal molar fractions *x*_A_^′^ = 0.04–0.06) were stored in desiccators before measurement to prevent interference from water traces in the FTIR spectra. Each sample was prepared by placing 10 μL of the LC mixture between KBr plates separated with plastic spacers of approximate thickness 50 μm. Infrared spectra were recorded between wavenumbers 800 and 4000 cm^–1^. The FTIR spectral data were used to quantify the concentrations of monomeric and dimeric acid species in the 5CB mixtures by analyzing the carbonyl stretching signals between 1630 and 1760 cm^–1^. It was verified the absence of large IR absorption signals of 5CB in the studied interval of wavenumbers. For aminobenzoic acids, the carbonyl signals are located at lower wavenumbers than those for the rest of the dopant acids,^22^ and a small signal of the 5CB between 1680 and 1700 cm^–1^ interferes with the carbonyl bands. This interference for the carbonyl signals of aminobenzoic acids (**1**–**4**) is corrected through baseline subtraction.

The carbonyl signals were fitted with a sum of two pseudo-Voigt functions (weighted sum of a Gaussian and Lorentzian peak functions)

1 $$y=\sum_{i=1}^{2}A_{i}\left[\frac{m_{i}}{\sigma_{\mathrm{Gi}}\sqrt{2\pi }}\cdot \exp\left(-\frac{{\left(\mathrm{\nu ̃}-{\mathrm{\nu ̃}}_{\mathrm{ci}}\right)}^{2}}{2\sigma_{\mathrm{Gi}}^{2}}\right)+\frac{1-m_{i}}{\pi }\cdot \frac{2\sigma_{\mathrm{Li}}}{4{\left(\mathrm{\nu ̃}-{\mathrm{\nu ̃}}_{\mathrm{ci}}\right)}^{2}+\sigma_{\mathrm{Li}}^{2}}\right]$$

where the subindex *i* = 1 in the peak center *ν̃*_*c*_, area *A*, Gaussian weight *m*, Gaussian width σ_*G*_, and Lorentzian width σ_*L*_ refers to the peak due to monomeric carbonyls. For carbonyl groups in dimers, each of the mentioned parameters is tagged with subindex *i* = 2.

### Computational Details

Quantum chemical calculations were performed to assess the energetics of three plausible dimers of 4-aminobenzoic acid depicted in Figure 5. All calculations have been performed with the Resolution-of-Identity second-order Møller–Plesset perturbation theory (RI-MP2),^23–25^ in conjunction with the all-electron cc-pVTZ basis set.^26^ Geometry optimizations were tightly converged and minimal structures were corroborated by means of numerical frequency calculations, where the Hessian is obtained by numerical differentiation of the analytical gradient. Perturbation theory calculations were sped up by using the RI-J approximation for the Coulomb integrals and the numerical chain-of-sphere integration for the Hartree–Fock exchange integrals,^27^ together with their corresponding auxiliary basis sets.^28,29^ Solvent effects were included by employing the Conductor-like Polarizable Continuum Model using 5CB as solvent with an average dielectric constant of 10.7.^30^ All calculations were performed with the ORCA quantum chemical package.^31^

## Results and Discussion

The functionalized benzoic acids used as dopants in mixtures with the nematic liquid crystal 5CB are shown in Figure 1. These aromatic acids have planar and rigid molecular structures. Even though liquid-crystalline phases have been reported for some *para*-substituted benzoic acids with alkyl,^32^ alkoxy,^33^ or alkylthio groups having chains of 4 or more carbon atoms,^34^ none of the substituted benzoic acids reported in the present study (**1**–**20**) exhibit mesomorphism. All of the mixtures of 5CB doped with functionalized benzoic acids exhibit marble and schlieren textures (Figure 2) when observed under a polarized optical microscope. These are the same textures exhibited by the pure 5CB, indicating that the nematic phase of 5CB remains unaltered after doping with these acids.^1^

**Figure 2:**
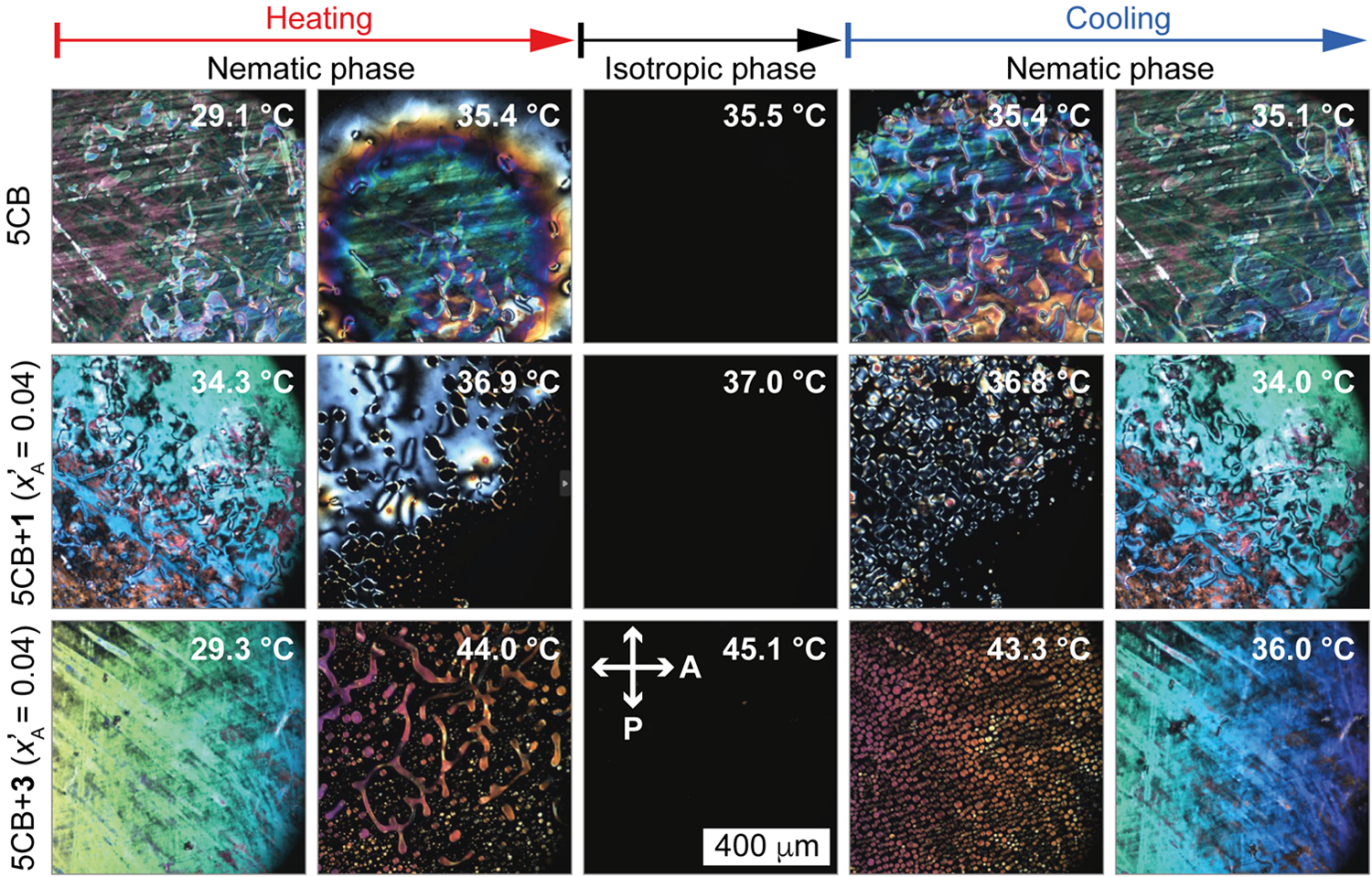
Polarized optical microscopy photographs showing the heating process (left), the nematic–isotropic transition (center), and the cooling of the samples (right) to return to the nematic phase. The first row of micrographs displays the nematic–isotropic transitions of pure 5CB. The second row displays the transitions of a mixture 5CB + 2-aminobenzoic acid (1) with nominal molar fraction *x*_A_^′^ = 0.04. The third row displays the transitions of a mixture 5CB + 4-aminobenzoic acid (3) with nominal molar fraction *x*_A_^′^ = 0.04. The arrows P and A display the orientations of the polarizer and analyzer, respectively. The magnification was 10× in all micrographs.

The nematic–isotropic transition (or isotropization) enthalpy of each 5CB + dopant mixture was measured by differential scanning calorimetry (DSC) at three levels of concentration for each dopant (molar fractions in the interval 0.005–0.06). For each mixture of 5CB + dopant, the average and standard deviation of the nematic–isotropic transition enthalpy are reported in Table 1. Each measured enthalpy of isotropization is like that of the pure 5CB (Δ_NI_ *H* = (0.55 ± 0.01) kJ/mol), implying that the dopant acids do not significantly affect the energetics of the nematic–isotropic transition. Since the magnitude of the transition enthalpy is directly related to the attractive interactions between 5CB molecules, the fact that these enthalpies do not alter on doping the LC with functionalized benzoic acids indicates that interactions between mesogenic molecules are not modified at low dopant concentrations.

**Table 1:** Nematic-Isotropic Transition Enthalpies Δ_NI_ *H* for 5CB Doped with Substituted Benzoic Acids **1**–**20** ^a^

dopant	Δ_NI_ *H*/(kJ/mol)	dopant	Δ_NI_ *H*/(kJ/mol)
**1**	0.53 ± 0.01	**11**	0.52 ± 0.02
**2**	0.58 ± 0.02	**12**	0.53 ± 0.03
**3**	0.59 ± 0.01	**13**	0.57 ± 0.03
**4**	0.57 ± 0.01	**14**	0.50 ± 0.04
**5**	0.55 ± 0.02	**15**	0.51 ± 0.03
**6**	0.56 ± 0.02	**16**	0.50 ± 0.02
**7**	0.52 ± 0.02	**17**	0.57 ± 0.01
**8**	0.55 ± 0.02	**18**	0.51 ± 0.02
**9**	0.56 ± 0.02	**19**	0.52 ± 0.04
**10**	0.524 ± 0.003	**20**	0.52 ± 0.02

a The reported enthalpies are averages computed from the results at three levels of dopant concentration. The uncertainties are the standard deviations of these measurements.

The nematic–isotropic transition temperature of a 5CB + dopant mixture (*T*_NI_) changes with respect to that of the pure 5CB (*T*_NI_^*^). This change of temperature is defined as Δ*T* = *T*_NI_ – *T*_NI_^*^. Figure 3 displays the changes Δ*T* as a function of dopant concentration (the numeric data are available in Supporting Information S3). These results indicate that some dopants like 4-aminobenzoic acid (**3**) or 4-fluorobenzoic acid (**10**) increase the nematic–isotropic transition temperature, yielding positive Δ*T*, while other dopants like 2-bromobenzoic acid (**5**) or 3-iodobenzoic acid (**12**) decrease the isotropization temperature.

**Figure 3:**
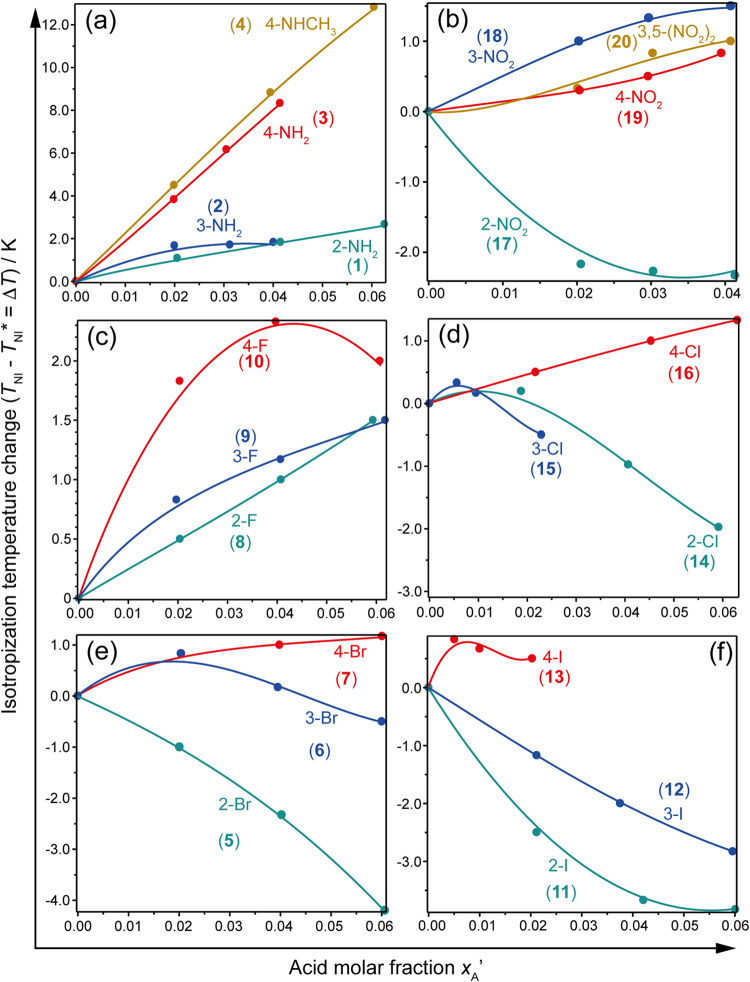
Experimental and modeled changes of the nematic–isotropic transition temperature of mixtures of 5CB liquid crystal doped with substituted benzoic acids. The fitting model is eq ([Disp-formula eq14]) with the ordering factor in eq ([Disp-formula eq15]) as a function of the monomeric and dimeric concentrations expressed in eqs ([Disp-formula eq5]) and ([Disp-formula eq6]). The fitting parameters are reported in Table 2. The information is displayed by families of dopants: (a) aminobenzoic acids, (b) nitrobenzoic acids, (c) fluorobenzoic acids, (d) chlorobenzoic acids, (e) bromobenzoic acids, and (f) iodobenzoic acids.

Examining the results displayed in Figure 3 leads to infer that the group of dopants, aminobenzoic acids, produces large positive changes of the nematic–isotropic transition temperature Δ*T*, with outstanding increments of approximately 8 and 13 °C for 4-aminobenzoic acid (**3**) and 4-*N*-methylaminobenzoic acid (**4**) at their maximum studied concentrations, respectively. Differently, the halogen and nitro-substituted benzoic acids confer subtle increments of Δ*T* to the doped liquid crystals; besides, they produce negative values of Δ*T* (decrease the *T*_NI_). The dopants 2-bromobenzoic (**5**) acid and 2-iodobenzoic acid (**11**) induced the most negative Δ*T* values of approximately −4 °C each. These findings suggest that for benzoic acid dopants dissolved in a nematic solvent like 5CB, the electron-withdrawing traits of the substituents and their position in the aromatic ring have an important influence on nematic–isotropic temperature shifts.

The change of the nematic–isotropic transition temperature indicates changes of the molecular order in the nematic phase. Although some minimum molecular order prevails upon slightly surpassing the nematic–isotropic transition temperature, as suggested by the pretransitional effects observed on the dielectric properties of polar nematogens like 5CB,^35^ the molecular order of a nematic liquid crystal S can be described in practical terms by the Haller model

$$S={\left(1-\frac{T}{T_{\mathrm{NI}}}\right)}^{\beta }>0;{T<T}_{\mathrm{NI}}$$

2 $$S=0;T\geq T_{\mathrm{NI}}$$

where β is a constant. For pure 5CB, *T*_NI_^*^ = 308.65 K. At *T* ≥ *T*_NI_^*^, S=0 , the molecular orientational order vanishes and the pure 5CB becomes isotropic. However, if the nematic-isotropic transition temperature of a mixture of 5CB + substance increases with respect to that of the pure 5CB (*T*_NI_(mix)>*T*_NI_^*^), this implies that at *T* = *T*_NI_^*^, the mixture of 5CB + substance has orientational order and S>0 , thus, the dopant substance enhances the molecular order of the mixture. Conversely, if mixing 5CB with a dopant causes a decreasing of the nematic–isotropic temperature, it means the dopant has a deleterious effect on the molecular order of the nematic phase. This is the most common case in mixtures of liquid crystals with nonmesogenic substances.

The change of *T*_NI_ (Δ*T* = *T*_NI_ – *T*_NI_^*^) can be used as an experimental quantity to trace the molecular order enhancement or reduction produced by a dopant in a nematic solvent. The increasing of Δ*T* is associated to order-enhanced nematic mixtures of 5CB + dopant, and on the contrary, decreased Δ*T* means less ordered nematic mixtures. The capability of a benzoic acid dopant to increase or decrease the molecular order depends on its molecular geometry and the extent of dimerization. It is known that carboxylic acids dimerize when dissolved in nonpolar solvents. This dimerization process (Scheme 1) produces molecular complexes with varied geometries depending on the molecular structure of the acids. It has been shown that molecularly rigid aromatic acids without substitutions increase the molecular order in their mixtures with 5CB.^19^ The dimerization process can be demonstrated by infrared spectroscopy: the carbonyl signal of the acids shifts to lower wavenumbers (frequencies) when the carboxylic groups interact through hydrogen bonds in the dimers.^36^ The dimerization of the acids also depends on the electron-withdrawing or donating effect of the substituents.^36,37^

**Scheme 1:**
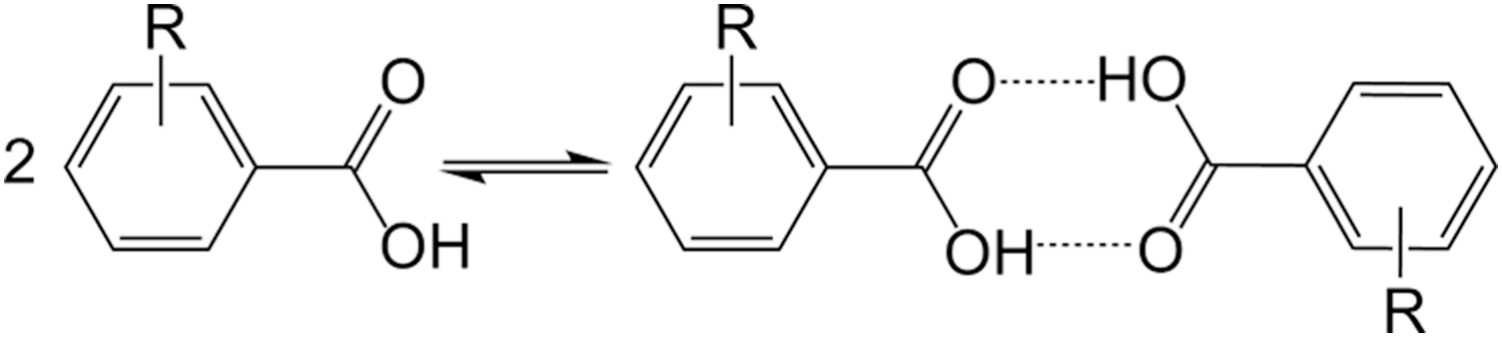
Dimerization of a Functionalized Benzoic Acid through Hydrogen Bonding^a^

a R is a functional group attached to the aromatic ring at an arbitrary position.

Dimerization of carboxylic acids is promoted in nonpolar solvents. The 5CB is considered a polar liquid crystal having a molecular dipole moment μ = 5*D*. However, the dimerization process of the benzoic acids is not expected to be impeded in 5CB, given that polar LC molecules in nonferroelectric nematic phases correlate antiparallelly, decreasing the polarity of the liquid crystal, a fact that has been observed through the strong pretransitional (isotropic to nematic) dielectric effect presented in 5CB.^35^

The acid dimerization in the 5CB liquid crystal was quantitatively assessed by numerical deconvolution of the infrared signals assigned to carbonyl (Figure 4). The carbonyl signals were fitted with a sum of two pseudo-Voigt functions (eq ([Disp-formula eq1])), as described in the Experimental section. In the infrared spectra of 5CB doped with aminobenzoic acids (dopants **1–4**, refer to Figure 4), the large red deconvoluted peaks are assigned to hydrogen-bonded carbonyls. This finding indicates that electron-donating groups like NH_2_ favor the dimerization of the acids. On the contrary, electron-withdrawing groups, such as halogens and nitro substituents, make these carboxylic substances more acidic and thus hinder their dimerization.^38^

**Figure 4:**
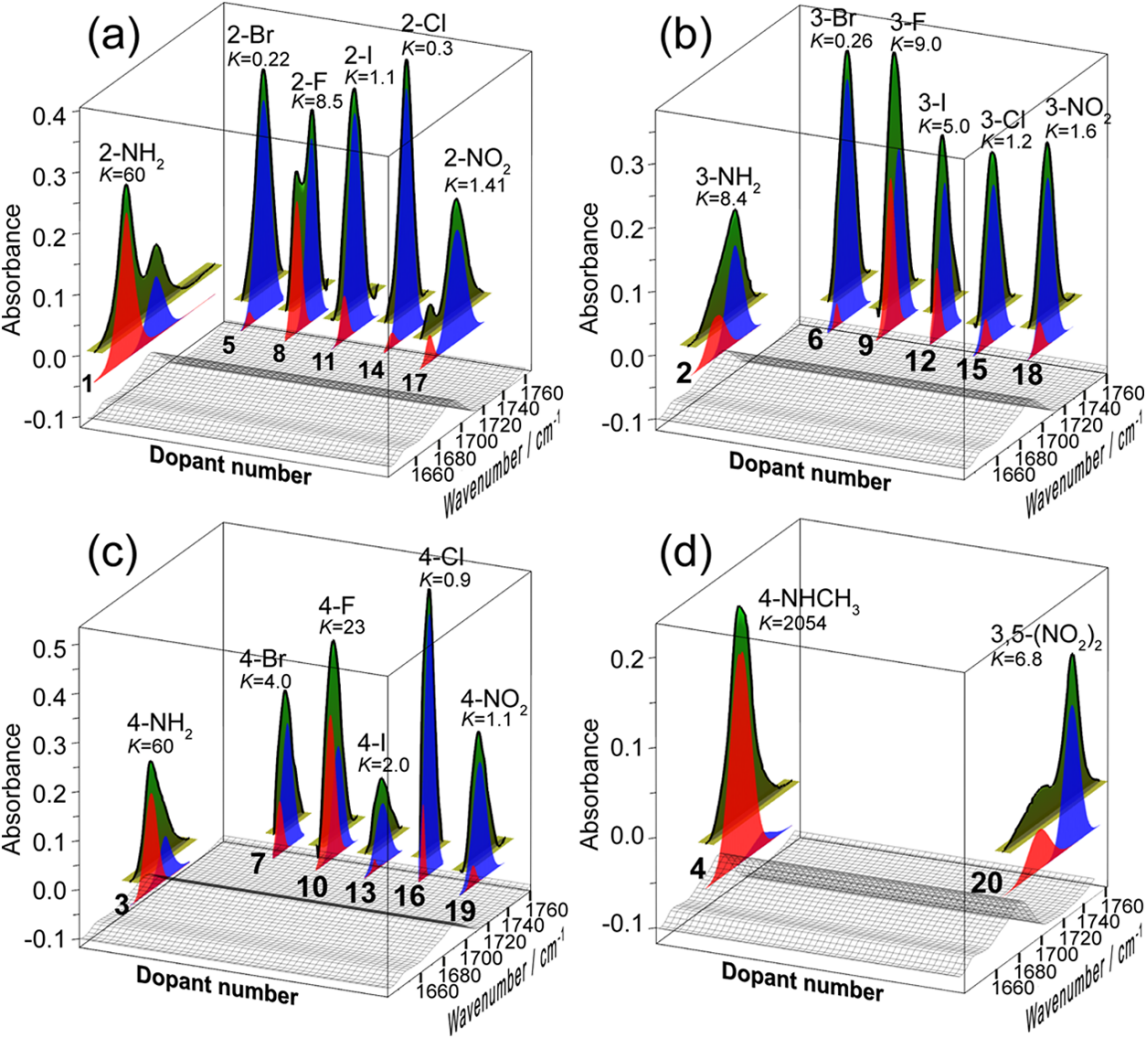
Infrared spectra of LC mixtures of 5CB + substituted benzoic acid dopants (**1**–**20**) and calculated equilibrium constants *K* for the dimerization of the dopant acids at 25 °C. The green bands are the experimental signals; the blue and red bands are obtained by deconvolution using nonlinear fitting with pseudo-Voigt functions. The blue bands with areas *A*_1_ are assigned to free carbonyls (not forming dimers through hydrogen bonding), while the red bands with areas *A*_2_ are assigned to carbonyls interacting through hydrogen bonds in dimers of the acids. (a) Benzoic acids substituted in *ortho* positions. (b) Benzoic acids substituted in *meta* positions. (c) Benzoic acids substituted in *para* positions. (d) Dopants 4-*N*-methylaminobenzoic acid and 3,5-dinitrobenzoic acid. The 5CB signal surface was artificially rendered in three dimensions by leaving the dopant number as a free variable. The small signal of the 5CB that interferes with the carbonyl signal of the amino-substituted dopants is subtracted through baseline corrections.

For benzoic acids with amino substitutions, besides the dimerization through hydrogen bonding connecting carboxylic groups (like in Scheme 1 and Figure 5(a)), other possibilities are the H-bonding linking the carboxylic and the amino groups (structure in Figure 5(b)), or the dimerization through anion–cation electrostatic interactions (Figure 5(c)). These possibilities were theoretically assessed for 4-aminobenzoic acid. The energies of the optimized structures of molecular complexes in Figure 5 were theoretically computed through quantum chemical methods (see details in Experimental methods). The optimization of the plausible structures of Figure 5 was performed considering their solvation in a medium of static dielectric constant of 10.7 (average value for 5CB at 25 °C calculated from the anisotropic dielectric constants, $\varepsilon_{\mathrm{average}}=\frac{2}{3}\varepsilon_{⊥}+\frac{1}{3}\varepsilon_{∥}$ ).^39^ The probability for each kind of complex was also computed through the Boltzmann distribution ( $\mathrm{probability}\left(i\right)=e^{-E_{i}/k_{B}T}/\sum e^{-E_{i}/k_{B}T}$ ). According to these results, the formation of asymmetric dimers or ionic complexes is improbable and will not be further considered.

**Figure 5:**
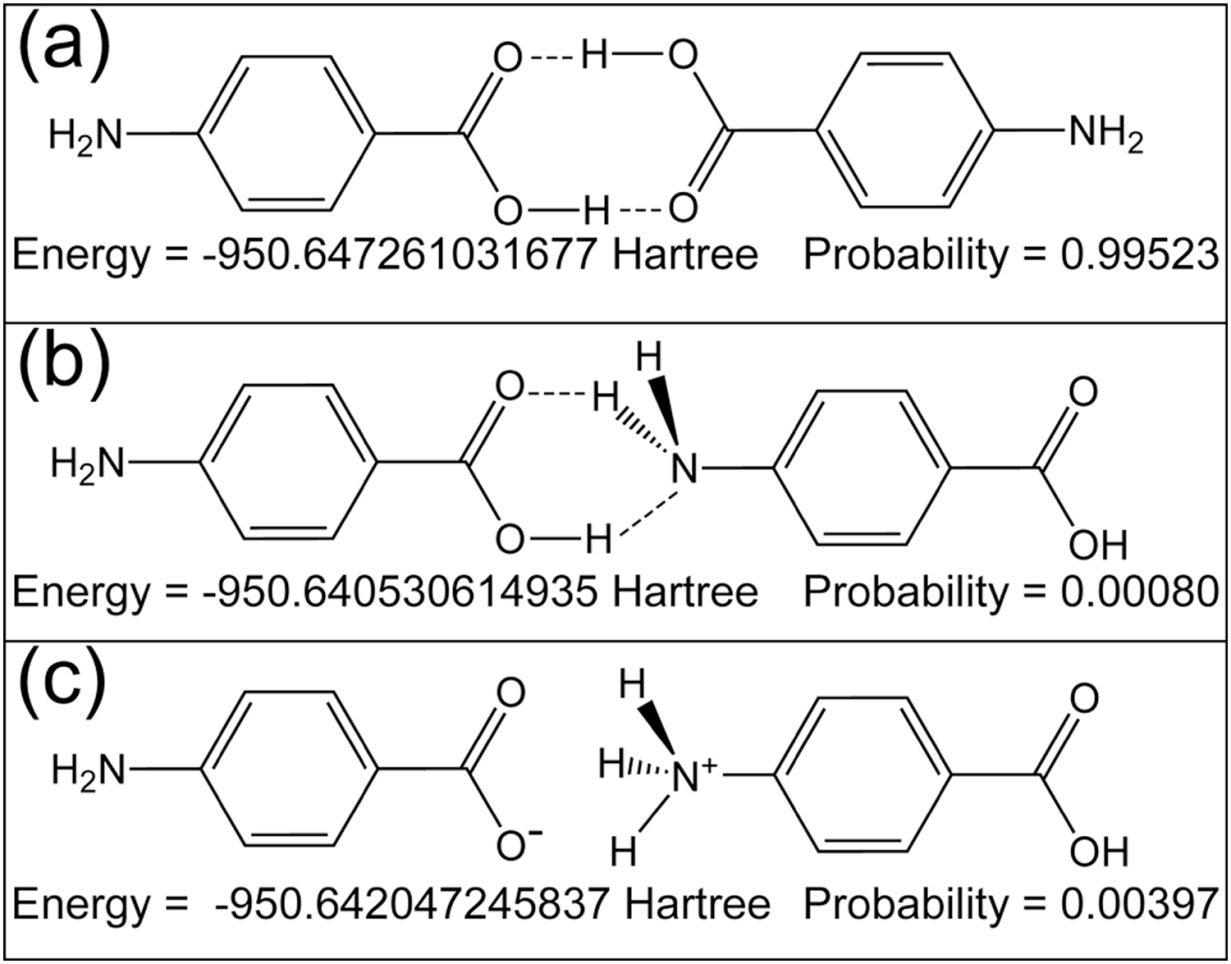
Possible dimerization mechanisms for 4-aminobenzoic acid. (a) Symmetric dimer through H-bonding of carboxylic groups. (b) Asymmetric dimer through H-bonding of the carboxylic group and amino groups. (c) Ionic interaction. The calculated energy and the associated probability for each dimer are reported.

The dimerization process of Scheme 1 was modeled through chemical equilibrium. The dissolution of a quantity *n*_A_^′^ (in mol units) of a benzoic acid A with an amount *n*_5CB_ of 5CB is characterized by the nominal (or preparation) mole fraction *x*_A_^′^ = *n*_A_^′^/(*n*_A_^′^ + *n*_5CB_). As the acid can dimerize in the mixture, quantities *n*_A_ and *n*_A2_ of monomeric and dimeric benzoic acid species emerge. The actual mole fractions of the monomeric and dimeric forms of the acid are represented by *x*_A_ and *x*_A2_, respectively. The relation between the nominal concentration of the acid and the actual concentration of monomers and dimers is (see proof in the Supporting Information)­

3 $$x_{A}^{\prime }=\frac{x_{A}+2x_{A2}}{1+x_{A2}}$$

Assuming the thermodynamic ideality of the system, the equilibrium constant for the dimerization process in Scheme 1 is

4 $$K=\frac{x_{A2}}{x_{A}^{2}}$$

The mole fraction of monomers and dimers of the benzoic acids can be solved from eqs ([Disp-formula eq3]) and ([Disp-formula eq4])­

5 $$x_{A}=\frac{1}{2K}\cdot \frac{\sqrt{4Kx_{A}^{\prime }\left(2-x_{A}^{\prime }\right)+1}-1}{2-x_{A}^{\prime }}$$

6 $$x_{A2}=\frac{1}{4K}\cdot {\left(\frac{\sqrt{4Kx_{A}^{\prime }\left(2-x_{A}^{\prime }\right)+1}-1}{2-x_{A}^{\prime }}\right)}^{2}$$

The areas of the modeled infrared signals of carbonyls are used to quantify the concentrations of monomers and dimers. It is assumed a proportionality between the concentrations *x*_A_ and *x*_A2_ with the peak areas *A*_1_ and *A*_2_ assigned to monomers and dimers, respectively (Beer law)

7 $$A_{1}={\mathrm{cx}}_{A}$$

8 $$A_{2}=2{\mathrm{cx}}_{A2}$$

The solution of the system of eqs ([Disp-formula eq5])–([Disp-formula eq8]) allowed the computation of the association constant *K* using the deconvoluted areas *A*_1_ and *A*_2_

9 $$K=\frac{r}{2x_{A}^{\prime }}\left[r\left(1-\frac{x_{A}^{\prime }}{2}\right)+1\right]$$

where *r* = *A*_2_/*A*_1_ is the ratio of deconvulated peak areas (dimer/monomer). The values of the dimerization equilibrium constants are reported in Figure 4. Among the studied dopants, the amino-substituted benzoic acids show the largest values of the association constants *K*. The electron-donating effect of the amino groups causes the aminobenzoic acids to be less acidic than the benzoic acids substituted with electron-withdrawing halogen or nitro groups. The low acidity of the aminobenzoic acids promotes their dimerization. On the contrary, a large acidity favors the ionization, diminishing the possibility of dimerization.

Several researchers have used thermodynamic models to explain the influence of dopants in liquid crystal mixtures in the nematic–isotropic transition temperature.^40,41^ Thermodynamically, the nematic–isotropic transition of the LC mixtures of 5CB + dopant takes place when the chemical potentials of the solvent 5CB in their nematic and isotropic phases even up (μ_5CB,N_ = μ_5CB,I_). Assuming the ideal thermodynamic behavior, the chemical potential of 5CB in the isotropic state of the mixtures is expressed as

10 $$\mu_{5\mathrm{CB},I}=\mu_{5\mathrm{CB},I}^{*}+\mathrm{RT}\ln x_{5\mathrm{CB}}$$

where μ_5CB,I_^*^ is the chemical potential of the pure isotropic solvent 5CB, *R* is the universal gas constant, *T* is the temperature, and *x*_5CB_ is the molar fraction of the solvent 5CB. A similar equation for the chemical potential of the nematic phase can be stated; however, since the nematic–isotropic transition temperature can increase or decrease depending on the enhancement or weakening of molecular order induced by the dopant, an *ordering factor* of the dopant should be considered (see Figure 6).

**Figure 6:**
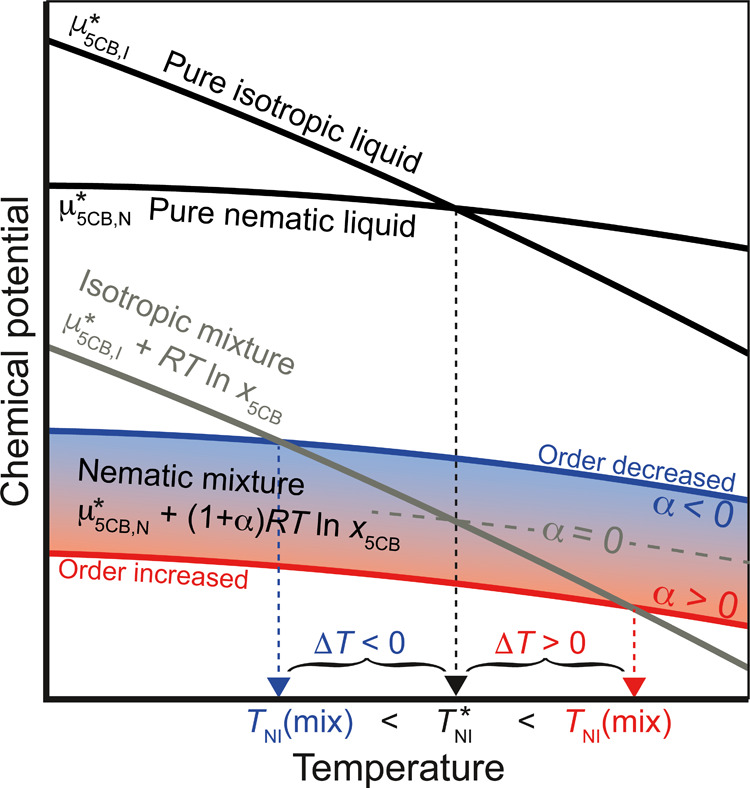
Sketch of the theoretical temperature variation of the chemical potential of pure solvent 5CB in nematic liquid crystal state (μ_5CB,N_^*^), pure solvent 5CB in isotropic liquid state (μ_5CB,I_^*^), solvent 5CB in a mixture with a dopant in nematic liquid crystal state (μ_5CB,N_ = μ_N_^*^ + (1 + α)*RT* ln *x*_5CB_), and solvent 5CB in a mixture in isotropic liquid state (μ_5CB,I_ = μ_I_^*^ + *RT* ln *x*_5CB_). In the doped nematic liquid crystals, the ordering factor α shifts up or down the chemical potential, changing the intersection coordinates of the nematic–isotropic transition. An ordering factor α < 0 yields a decreasing of the nematic–isotropic transition temperature. Conversely, an ordering factor α > 0 leads to an increasing of the nematic–isotropic transition temperature. An ordering factor α = 0 leaves the nematic–isotropic transition unchanged with respect to that of the pure nematic solvent.

The following expression for the chemical potential of the nematic solvent 5CB in the mixtures (5CB + dopant) is proposed

11 $$\mu_{5\mathrm{CB},N}=\mu_{5\mathrm{CB},N}^{*}+\left(1+\alpha \right)\mathrm{RT}\ln x_{5\mathrm{CB}}$$

where μ_5CB,N_^*^ is the chemical potential of the pure 5CB in the nematic LC state, and α is an ordering factor. The ordering factor shifts up or down the chemical potential of the nematic mixtures, producing negative changes of *T*_NI_ if α < 0, or positive changes if α > 0 (refer to Figure 6 for a graphical depiction of the transitions). In this model, if α = 0, the isotropization temperature remains the same as in the pure nematic liquid crystal solvent. At the nematic–isotropic transition temperature *T*_NI_, the chemical potentials of eqs ([Disp-formula eq10]) and ([Disp-formula eq11]) equalize (μ_5CB,N_ = μ_5CB,I_), implying that

12 $$\alpha R\ln x_{5\mathrm{CB}}=\frac{\Delta_{\mathrm{NI}}G}{T_{\mathrm{NI}}}$$

where Δ_NI_ *G* = μ_5CB,I_^*^ – μ_5CB,N_^*^ is the Gibbs energy change of the nematic–isotropic transition. Differentiating this equation with respect to the transition temperature *T*_NI_, and using the Gibbs–Helmholtz relation,^42^ the following equation arises

13 $$\alpha \ln x_{5\mathrm{CB}}=\frac{\Delta_{\mathrm{NI}}H}{R}\left(\frac{1}{T_{\mathrm{NI}}}-\frac{1}{T_{\mathrm{NI}}^{*}}\right)$$

where Δ_NI_ *H* is the enthalpy change of the nematic–isotropic transition and *T*_NI_^*^ is the isotropization temperature of the pure nematic solvent. Expressing the molar fraction of the solvent 5CB in terms of the molar fractions of the monomeric and dimeric forms of the acid solute, eq ([Disp-formula eq13]) can be rearranged as

14 $$\Delta T=T_{\mathrm{NI}}-T_{\mathrm{NI}}^{*}=-T_{\mathrm{NI}}^{*}\cdot \frac{c\alpha \ln\left(1-x_{A}-x_{A2}\right)}{1+c\alpha \ln\left(1-x_{A}-x_{A2}\right)}$$

The dimensionless quantity *c* merges several constants (*c* = *RT*_NI_^*^/Δ_NI_ *H*). As the ordering factor α can change with the concentration of dopants, a linear combination is proposed as an ansatz

15 $$\alpha =\alpha_{0}+\alpha_{1}x_{A}+\alpha_{2}x_{A2}$$

with α_0_, α_1_, and α_2_ as fitting parameters. The monomer and dimer molar fractions are expressed in eqs ([Disp-formula eq5]) and ([Disp-formula eq6]) as functions of the nominal dopant molar fraction *x*_A_^′^. The model eq ([Disp-formula eq14]) with the ordering factor expressed in eq ([Disp-formula eq15]) was used to fit the changes of the nematic–isotropic transition temperature of the mixtures of 5CB + substituted benzoic acids (Figure 3 and Table 2).

**Table 2:** Fitting Parameters of the Ordering Factor α Variation (α_0_, α_1_, and α_2_) Computed by Nonlinear Fitting of the Nematic–Isotropic Transition Temperature Changes Measured for the Mixtures of 5CB with Substituted Benzoic Acids^a^

dopant	substituent R	α_0_	α_1_	α_2_	*s*(fit)/K	α(*x*_A_^′^ = 0.01)
**11**	2-I	–0.0980	0.857	2.03	0.088	–0.0894
**17**	2-NO_2_	–0.0957	1.25	4.66	0.123	–0.0829
**12**	3-I	–0.0381	–0.318	1.46	0.017	–0.0404
**5**	2-Br	–0.0312	–0.152	–10.1	0.035	–0.0330
**20**	3,5-(NO_2_)_2_	–0.0068	1.97	–5.33	0.066	0.0079
**19**	4-NO_2_	0.0112	–0.191	7.46	0.008	0.0101
**15**	3-Cl	0.0716	–7.76	144	0.038	0.0123
**14**	2-Cl	0.0279	–1.64	38.2	0.080	0.0129
**16**	4-Cl	0.0163	–0.00654	–0.370	0.003	0.0162
**8**	2-F	0.0177	0.0433	0.132	0.003	0.0182
**7**	4-Br	0.0421	–0.980	2.07	0.049	0.0337
**9**	3-F	0.0427	–0.959	0.998	0.041	0.0352
**18**	3-NO_2_	0.0337	0.290	–8.69	0.017	0.0353
**6**	3-Br	0.0552	–1.89	55.7	0.105	0.0378
**1**	2-NH_2_	0.0550	–1.04	0.366	0.035	0.0496
**13**	4-I	0.169	–16.5	218	0.080	0.0520
**2**	3-NH_2_	0.0790	–1.02	–2.41	0.109	0.0685
**10**	4-F	0.0871	–0.626	–2.35	0.098	0.0794
**3**	4-NH_2_	0.121	8.79	–4.21	0.048	0.164
**4**	4-NHCH_3_	0.229	42.8	–4.65	0.041	0.271

a The goodness of the fittings is assessed through the standard deviation of the fit (*s*(fit)), which expresses the mean error of Δ*T* produced by the models. The ordering factor at the nominal molar fraction of dopant *x*_A_^′^ = 0.01 is computed to compare the ordering effect of the dopants at the same concentration. The list is organized from the most disordering dopant to the most ordering one.

The ordering factors at *x*_A_^′^ = 0.01 displayed in Table 2 are listed from the most disorder-inducing dopants to the most order-enhancing ones. Among benzoic acids, those with electron-withdrawing substitutions in *ortho* or *meta* positions bear the most order-destroying effect (2-iodobenzoic acid, 2-nitrobenzoic acid, for instance). In contrast, dopants like 4-*N*-methylaminobenzoic acid and 4-aminobenzoic acid, having electron-donating groups in *para* positions, bestow the highest order-enhancing effect on the liquid crystal mixtures. From our knowledge, the dopant **4** (4-*N*-methylaminobenzoic acid) has the top order-enhancing effect from a nonmesogen at low concentrations reported to date, yielding an outstanding nematic–isotropic temperature increment of 12 K with a modest nominal concentration of acid *x*_A_^′^ = 0.06. However, doping 5CB with 4-*N*-methylaminobenzoic acid at concentrations beyond *x*_A_^′^ = 0.06 confronts a limit imposed by the substance’s solubility.

In Figure 7, a general trend regarding the influence of the substitution position of the group in the aromatic ring of the benzoic acids on the ordering factor can be inferred: *ortho* ≈ *meta* < *para*. The linear geometry of the dimerized benzoic acids with *para* (position 4) substitutions implies larger ordering factors than those obtained with *ortho* (position 2) and *meta* (position 3) substitutions. Moreover, the logarithm of the association constant *K* (log *K*, which is proportional to the Gibbs energy decrease upon dimerization) shows a linear correlation (*r* ^2^ = 0.95) with the ordering factor in the benzoic acids with *para* substitutions, which is absent in the *ortho-* and *meta-*substituted acids (see Figure 7(b)). This trend indicates that the dimerization is favored for the *para*-substituted acids in the liquid-crystalline solvent, pointing to a synergetic effect between the molecular association of the *para-*substituted acids and the molecular ordering of the nematic environment. Interestingly, for the substituted benzoic acids, the general trend of their ordering factor as a function of the substitution position (*ortho* ≈ *meta* < *para*) is like that of their melting points for each substituent group (*ortho* < *meta* < *para*, refer to data in Figure 1, see the inset in Figure 7(a)). The melting points of the dopants increase with the hydrogen-bonding capability of the molecules and with the enhanced molecular packing attained with the elongated dimers of the *para*-substituted benzoic acids. These same factors manifest in the nematic ordering capabilities of the benzoic acids as dopants in the nematic environment of the LC 5CB.

**Figure 7:**
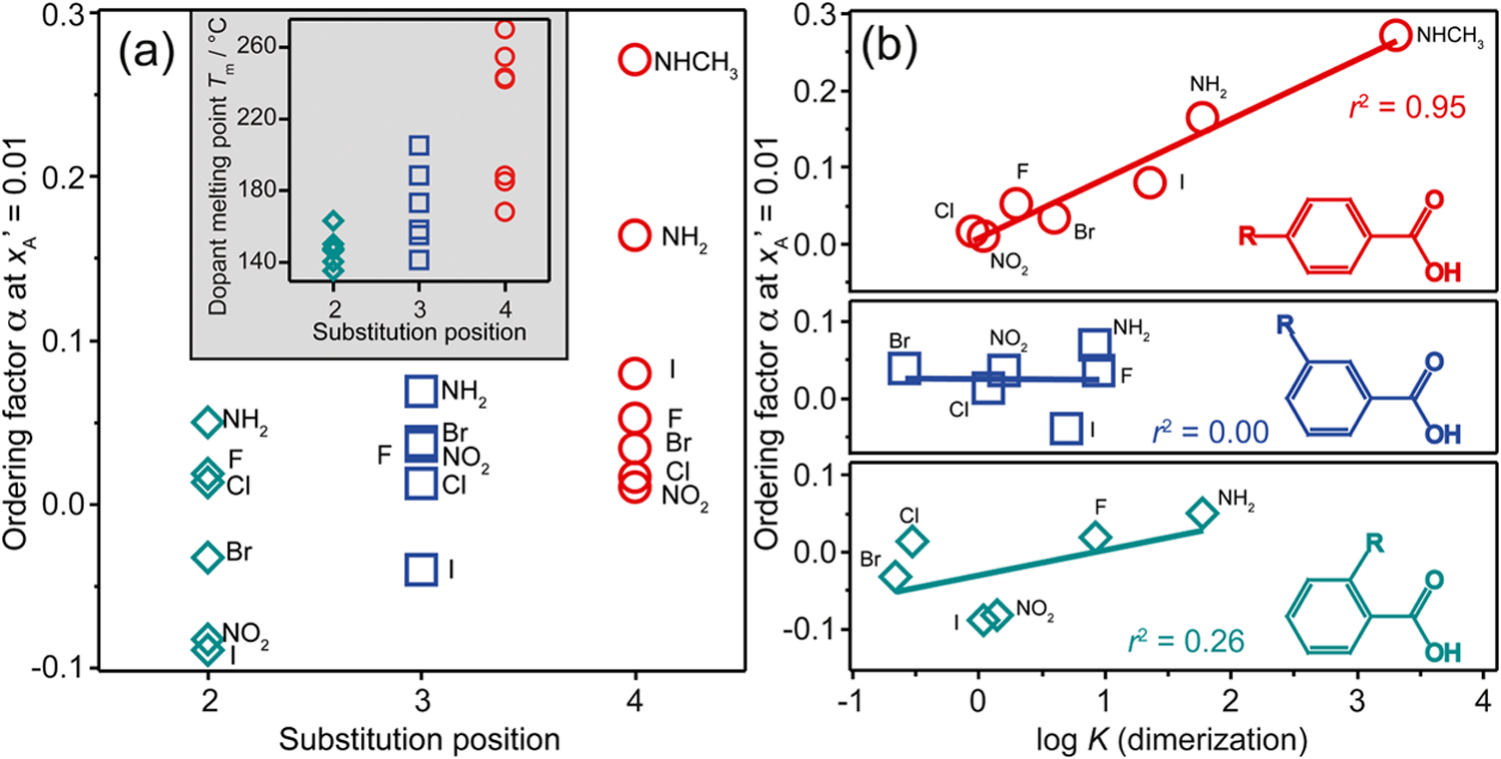
Structure–property relations of the ordering factor α. (a) Ordering factor α calculated at the nominal concentration of dopant acid *x*_A_^′^ = 0.01 as a function of the substitution position in the aromatic ring. The main trend of the structure–property relation is *ortho* ≈ *meta* < *para*. The inset plot (with gray background) shows the trend of melting points of the substituted benzoic acids as a function of the substitution position. (b) Ordering factor α calculated at the nominal concentration of dopant acid *x*_A_^′^ = 0.01 as a function of the logarithm of the dimerization equilibrium constant *K* (proportional to Gibbs energy of dimerization).

A better understanding of the influence of the molecular geometry of the dimers on the ordering of the nematic liquid crystal mixtures is achieved by inspecting the aspect ratio length/diameter (refer to Figure 8). The dimers of *p*-substituted acids have larger shape aspect ratios (length/diameter) than those dimers of acids with *o* and *m* substitutions (as depicted in Figure 8(a)). The calamitic shape of *p*-dimers fits better in the uniaxial nematic solvent, enhancing the molecular order of the mixture and consequently increasing the *T*_NI_.

**Figure 8:**
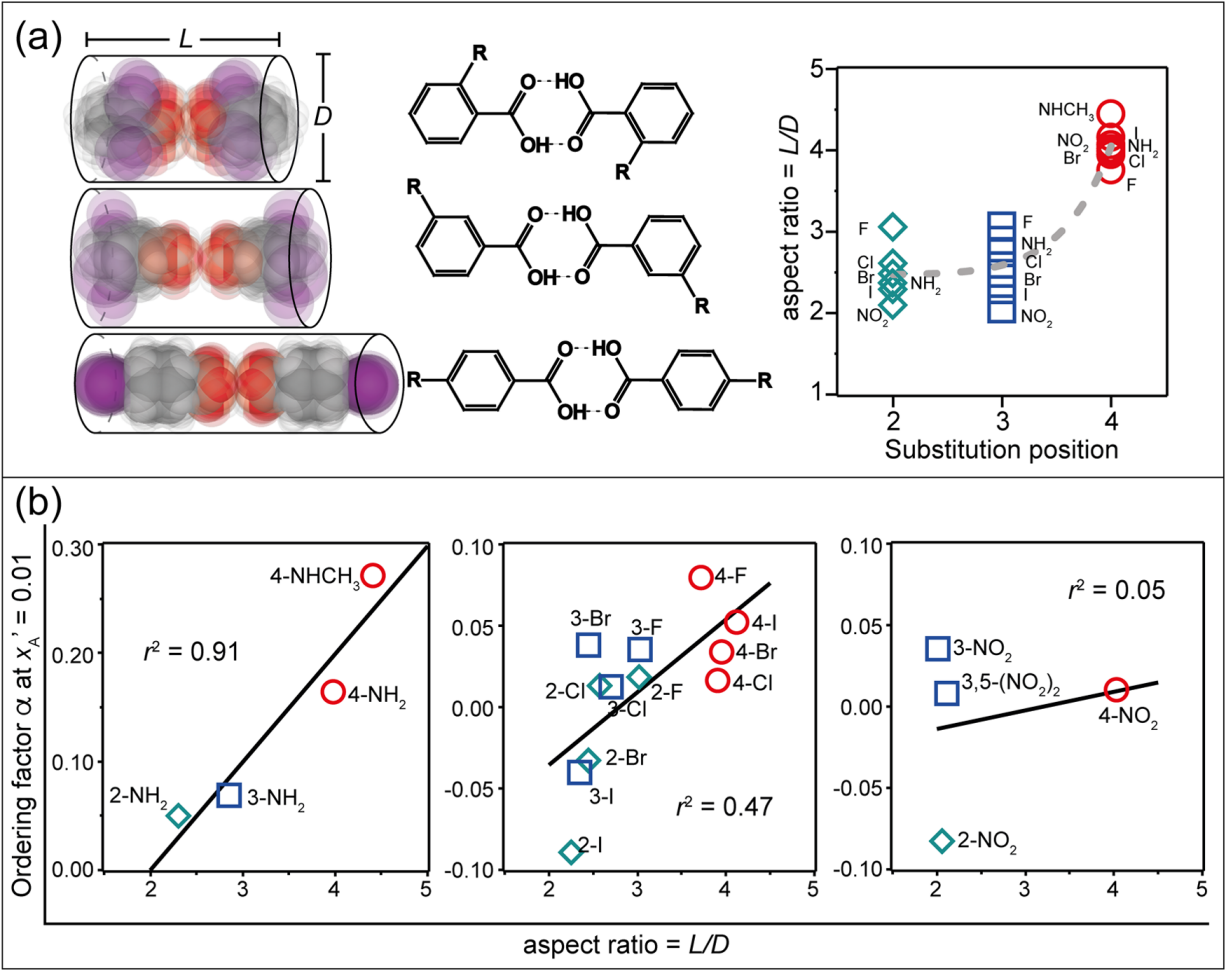
(a) Aspect ratio length/diameter of the substituted benzoic acid dimers; the dotted line roughly depicts the trend of aspect ratios: *ortho* ≈ *meta* < *para*. (b) Ordering factors at the nominal dopant concentration *x*_A_^′^ = 0.01 as a function of the dimer aspect ratios. Several ball models of dimers of *ortho-*, *meta-*, and *para*-substituted benzoic acids at varied rotations around the long molecular axis of the dimers were merged to sketch the cylindrical space occupied by these dimers aligned in a uniaxial environment. The aspect ratios of the cylinders were computed using reported crystal structures of the dopants.^43–57^ The distances were measured using software Vesta.^58^

The ordering factor reveals a correlation with the dimer aspect ratio for only aminobenzoic acids. This finding evidences that a purely molecular geometric argument only applies for carboxylic acids that quantitatively dimerize, with high spontaneity. For benzoic acids having electron-withdrawing substituents (halogen and nitro groups), dimers and monomers coexist, making invalid any explanation based exclusively on the molecular shape of dimers. The role of monomeric benzoic acids in the nematic order of the liquid crystal mixtures is mostly deleterious.

## Conclusions

A comprehensive analysis of 20 substituted benzoic acids used as dopants of the liquid crystal 5CB revealed the roles of the electronic effects and molecular geometry of the acids in varying the nematic–isotropic temperature transitions of the LC mixtures. Polarized optical microscopy imaging ratified that all of the mixtures of 5CB + substituted benzoic acid exhibit the nematic mesophase between 25 °C and their corresponding isotropization temperatures. The nematic–isotropic transition enthalpy of the mixtures measured by differential scanning calorimetry remained unchanged compared with that of the pure 5CB, hence proving that the intermolecular interactions between mesogens in the mixtures are basically unaltered upon doping with substituted benzoic acids. Dimerization of the acids through carboxyl–carboxyl hydrogen bonding is conceived as a key phenomenon underlying the nematic order enhancement of the LC mixtures. The dimerization of the carboxylic acids was evaluated by infrared spectroscopy through numerical deconvolution of the carbonyl signals. The degree of dimerization strongly depends on the electron-withdrawing or donating effects of the substituting groups; electron-donating groups like amino or methylamino favor the dimerization of the benzoic acids dissolved in the liquid crystal solvent, while electron-withdrawing halogen and nitro groups deter this molecular association. An ideal thermodynamic model (eq ([Disp-formula eq14])) was developed, yielding good fittings of the experimental data of nematic–isotropic transition temperature changes as a function of concentration of dopants. The derivation of this thermodynamic model is based on the chemical potential shifting of the liquid-crystalline solvent 5CB upon its mixture with the dopants. This thermodynamic model includes an *ordering factor α* as a fitting parameter, which correlates with the nematic order enhancement of the dopants. The aspect ratio length/diameter of the formed dimers was explored as a molecular geometric variable in the order alteration exerted by the dopants, leading to the conclusion that a purely geometrical argument based on the aspect ratio of acid dimers is insufficient to explain the order increasing or decreasing capabilities of the substituted benzoic acid dopants. This rationale based on the molecular geometry of the dimers is only valid for the aminobenzoic acids, which spontaneously dimerize in the 5CB solvent as indicated by their high dimerization equilibrium constants. In general, benzoic acids substituted at the *para* position (position 4) with an electron-donating group (such as amino or methylamino) endow large nematic order in mixtures with the 5CB liquid crystal. A slight doping of 5CB with 4-*N*-methylaminobenzoic acid (dopant **4**) at a mole fraction of 0.06 yields an astounding increment of the nematic–isotropic transition temperature of approximately 13 K, a breakthrough for a nonmesogen at minimum concentrations.

## Supplementary Material



## Notes

Details of DSC and infrared spectra calculations, data of nematic–isotropic transition temperatures, the structures of theoretically optimized dimers of 4-aminobenzoic acid, and a proof of the formula that correlates monomer, dimer, and nominal molar fractions (PDF)

## References

[1] Dierking, I. Textures of Liquid Crystals; John Wiley & Sons, 2003 10.1002/3527602054.

[2] Dunmur, D. ; Fukuda, A. ; Luckhurst, G. Physical Properties of Liquid Crystals: Nematics; Institution of Electrical Engineers, 2001.

[3] Singh, S. Handbook of Liquid Crystals—Vol. I: Foundations and Fundamental Aspects; Springer International Publishing, 2024. 10.1007/978-3-031-50058-9.

[4] Wu, P. C. ; Kumar, S. ; Lee, W. Introduction: From Conventional to Unconventional Liquid Crystals. In Unconventional Liquid Crystals and Their Applications; Lee, W. ; Kumar, S. , Eds.; De Gruyter, 2021 ; pp 1–108 10.1515/9783110584370.

[5] Khoo, I. C. Liquid Crystals; Wiley, 2022 10.1002/9781119705819.

[6] Lueder, E. ; Knoll, P. ; Lee, S. H. Liquid Crystal Displays: Addressing Schemes and Electro-Optical Effects; Wiley, 2022 10.1002/9781119667940.

[7] Jones J. C. (2018). The Fiftieth
Anniversary of the Liquid Crystal Display. Liq.
Cryst. Today.

[8] Hakemi H. (2017). Polymer-Dispersed
Liquid Crystal Technology ‘Industrial Evolution and Current
Market Situation. Liq. Cryst. Today.

[9] Kleman, M. ; Lavrentovich, O. D. Soft Matter Physics: An Introduction; Springer-Verlag: New York, 2003 10.1007/b97416.

[10] Yeh, P. ; Gu, C. Optics of Liquid Crystal Displays, 2nd ed.; Wiley: Hoboken, 2009.

[11] Mukherjee P. K. (1998). The Puzzle
of the Nematic–Isotropic Phase Transition. J. Phys.: Condens. Matter.

[12] Duran H., Gazdecki B., Yamashita A., Kyu T. (2005). Effect of Carbon Nanotubes
on Phase Transitions of Nematic Liquid Crystals. Liq. Cryst..

[13] Li F., Buchnev O., Cheon C. I., Glushchenko A., Reshetnyak V., Reznikov Y., Sluckin T. J., West J. L. (2006). Orientational
Coupling Amplification in Ferroelectric Nematic Colloids. Phys. Rev. Lett..

[14] Li F., Buchnev O., Cheon C. I., Glushchenko A., Reshetnyak V., Reznikov Y., Sluckin T. J., West J. L. (2007). Erratum:
Orientational Coupling Amplification in Ferroelectric Nematic Colloids
[Phys. Rev. Lett.97, 147801 (2006)]. Phys. Rev.
Lett..

[15] Lopatina L. M., Selinger J. V. (2009). Theory of Ferroelectric Nanoparticles
in Nematic Liquid
Crystals. Phys. Rev. Lett..

[16] Park J. W., Labes M. (1976). Broadening of the Nematic
Temperature Range by a Non-Mesogenic Solute
in a Nematic Liquid Crystal. Mol. Cryst. Liq.
Cryst..

[17] Vashchenko P. V., Minenko S. S., Vus K. O., Kolosova O. S., Cherniakova M. Y., Belikov K. N., Lisetski L. N. (2025). Enhancement of the nematic ordering
in cyanobiphenyl 5CB doped with salicylaldoxime due to supramolecular
nanostructuring. J. Mol. Liq..

[18] Vashchenko P. V., Brodskii R. Y., Gvozdovskyy I. A., Minenko S. S., Sofronov D. S., Nesterkina M., Lisetski L. N. (2026). Dopant-induced supramolecular nanostructuring
in hydrogen-bonded liquid crystals (HBLC): A specific case of nematic
5CB and benzene diols. J. Mol. Liq..

[19] Jirón V., Castellón E. (2020). Increased
Nematic–Isotropic Transition Temperature
on Doping a Liquid Crystal with Molecularly Rigid Carboxylic Acids. J. Phys. Chem. B.

[20] Sharma D. (2010). Non-isothermal
kinetics of melting and nematic to isotropic phase transitions of
5CB liquid crystal. J. Therm. Anal. Calorim..

[21] CRC Handbook of Chemistry and Physics, 95th ed.; Haynes, W. M. , Ed.; CRC Press: Boca Raton, 2014.

[22] Nummert V., Travnikova O., Vahur S., Leito I., Piirsalu M., Mäemets V., Koppel I., Koppel I. A. (2006). Influence of Substituents
on the Infrared Stretching Frequencies of Carbonyl Group in Esters
of Benzoic Acid. J. Phys. Org. Chem..

[23] Feyereisen M., Fitzgerald G., Komornicki A. (1993). Use of approximate integrals in ab
initio theory. An application in MP2 energy calculations. Chem. Phys. Lett..

[24] Weigend F., Häser M., Patzelt H., Ahlrichs R. (1998). RI-MP2: optimized
auxiliary
basis sets and demonstration of efficiency. Chem. Phys. Lett..

[25] Weigend F., Häser M. (1997). RI-MP2: First derivatives and global
consistency. Theor. Chem. Acc..

[26] Woon D. E., Dunning T. H. (1993). Gaussian-basis sets
for use in correlated molecular
calculations. 3. The atoms aluminum through argon. J. Chem. Phys..

[27] Helmich-Paris B., de Souza B., Neese F., Izsák R. (2021). An improved
chain of spheres for exchange algorithm. J.
Chem. Phys..

[28] Weigend F. (2006). Accurate Coulomb-fitting
basis sets for H to Rn. Phys. Chem. Chem. Phys..

[29] Weigend F., Köhn A., Hättig C. (2002). Efficient use of the correlation
consistent basis sets in {DF-MP2} and {DF-MP2-F12} calculations. J. Chem. Phys..

[30] Barone V., Cossi M. (1998). Quantum Calculation
of Molecular Energies and Energy Gradients in
Solution by a Conductor Solvent Model. J. Phys.
Chem. A.

[31] Neese F., Wennmohs F., Becker U., Riplinger C. (2020). The ORCA quantum
chemistry program package. J. Chem. Phys..

[32] Kato T., Jin C., Kaneuchi F., Uryu T. (1993). Effect of the Molecular Orientation
on the Stability of Hydrogen-Bonded Benzoic Acid Dimers. Infrared
Study of Liquid-Crystalline 4-Alkylbenzoic Acids. Bull. Chem. Soc. Jpn..

[33] Blumstein A., Patel L. (1978). Molecular Arrangement
in Mesophases of Some *p-n*-Alkoxybenzoic
Acids. Mol. Cryst. Liq. Cryst..

[34] Arakawa Y., Sasaki Y., Igawa K., Tsuji H. (2017). Hydrogen bonding liquid
crystalline benzoic acids with alkylthio groups: phase transition
behavior and insights into the cybotactic nematic phase. New J. Chem..

[35] Van
Roie B., Leys J., Denolf K., Glorieux C., Pitsi G., Thoen J. (2005). Weakly first-order character of the nematic-isotropic phase transition
in liquid crystals. Phys. Rev. E.

[36] Silverstein, R. M. ; Bassler, G. C. Spectrometric Identification of Organic Compounds, 7th ed.; John Wiley & Sons, Inc.: Hoboken, 2005.

[37] Sathya
Prabu N. P., Madhu Mohan M. L. N. (2013). Thermal Analysis of Hydrogen Bonded
Benzoic Acid Liquid Crystals. J. Therm. Anal.
Calorim..

[38] Wade, L. G. ; Simek, J. W. Química Orgánica, 9th ed.; Pearson Educación S.A. de C.V.: México, 2017.

[39] Thoms E., Yu L., Richert R. (2022). From very
low to high fields: The dielectric behavior
of the liquid crystal 5CB. J. Mol. Liq..

[40] Kronberg B., Gilson F. R., Patterson D. (1976). Effect of
Solute Size and Shape on
Orientational Order in Liquid Crystal Systems. J. Chem. Soc., Faraday Trans. 2.

[41] Sunohara K., Fujimaru A., Shinya A., Kobinata S. (1995). Phase Behavior of Nematic-Nonnematic
Binary Systems. Mol. Cryst. Liq. Cryst. Sci.
Technol., Sect. A.

[42] Atkins, P. ; De Paula, J. . Atkins’ Physical Chemistry, 8th ed.; Oxford University Press: Oxford, 2006.

[43] Çelik Í., Akkurt M., Necefoğlu H., Aybirdi Ö., García-Granda S. (2009). 4-(Methylamino)­Benzoic
Acid. Acta Crystallogr., Sect. E:Struct. Rep.
Online.

[44] Athimoolam S., Natarajan S. (2007). 4-Carboxyanilinium
(2 R,3 R)-Tartrate and a Redetermination
of the α-Polymorph of 4-Aminobenzoic Acid. Acta Crystallogr., Sect. C:Cryst. Struct. Commun..

[45] Portalone G. (2009). A Redetermination
of 2-Nitrobenzoic Acid. Acta Crystallogr., Sect.
E: Struct. Rep. Online.

[46] Boone C. D. G., Derissen J. L., Schoone J. C. (1977). Anthranilic Acid
II (o-Aminobenzoic
Acid). Acta Crystallogr., Sect. B: Struct. Crystallogr.
Cryst. Chem..

[47] Williams P. A., Hughes C. E., Lim G. K., Kariuki B. M., Harris K. D. M. (2012). Discovery
of a New System Exhibiting Abundant Polymorphism: M-Aminobenzoic Acid. Cryst. Growth Des..

[48] Hathwar V. R., Thakur T. S., Dubey R., Pavan M. S., Guru
Row T. N., Desiraju G. R. (2011). Extending the Supramolecular Synthon
Based Fragment Approach (SBFA) for Transferability of Multipole Charge
Density Parameters to Monofluorobenzoic Acids and Their Cocrystals
with Isonicotinamide: Importance of C–H···O,
C–H···F, and F···F Intermolecular
Regions. J. Phys. Chem. A.

[49] Mukherjee A., Desiraju G. R. (2014). Halogen Bonds in
Some Dihalogenated Phenols: Applications
to Crystal Engineering. IUCrJ.

[50] Domenicano A., Schultz G., Hargittai I., Colapietro M., Portalone G., George P., Bock C. W. (1990). Molecular
Structure
of Nitrobenzene in the Planar and Orthogonal Conformations: A Concerted
Study by Electron Diffraction, X-Ray Crystallography, and Molecular
Orbital Calculations. Struct. Chem..

[51] Nygren C. L., Wilson C. C., Turner J. F. C. (2005). On the
Solid State Structure of 4-Iodobenzoic
Acid. J. Phys. Chem. A.

[52] Fotović L., Bedeković N., Pičuljan K., Stilinović V. (2022). Order versus
Disorder in the Cocrystals of *m* -Halogenopyridines
with *m* -Halogenobenzoic Acids: The Effects of the
I···O Halogen Bond. Cryst. Growth
Des..

[53] Ndima L., Betz R. (2016). Redetermination of the Crystal Structure of 3-Bromobenzoic Acid,
C_7_H_5_BrO_2_. Z.
Kristallogr. - New Cryst. Struct..

[54] Kanters J. A., Kroon J., Hooft R., Schouten A., van Schijndel J. A., Brandsen J. (1991). Temperature-Dependent
Order-Disorder Phenomena in Crystal
Structures Containing Dimers of Carboxylic Acids: The Crystal and
Molecular Structure of 3,5-Dinitrobenzoic Acid at Room and Liquid
Nitrogen Temperature and Statistics of the Geometries of Hydrogen-Bonded
Carboxyl Groups. Croat. Chem. Acta.

[55] Groom C. R., Bruno I. J., Lightfoot M. P., Ward S. C. (2016). The Cambridge Structural
Database. Acta Crystallogr., Sect. B:Struct.
Sci., Cryst. Eng. Mater..

[56] Polito M., D’Oria E., Maini L., Karamertzanis P. G., Grepioni F., Braga D., Price S. L. (2008). The Crystal Structures
of Chloro and Methyl Ortho-Benzoic Acids and Their Co-Crystal: Rationalizing
Similarities and Differences. CrystEngComm.

[57] Liwporncharoenvong T., Luck R. L. (2002). The Effects, Assessed by Electrochemical Techniques
and Single Crystal Structures, of Ortho Substitution on Benzoate Ligands
Supporting the Quadruply-Bonded Dimolybdenum Bond. Inorg. Chim. Acta.

[58] Momma K., Izumi F. (2011). *VESTA 3* for Three-Dimensional
Visualization of Crystal,
Volumetric and Morphology Data. J. Appl. Crystallogr..

