# Supplementary material for: The Role of Functional Groups in Substituted Benzoic Acids Used as Dopants in Liquid Crystal Mixtures on the Nematic–Isotropic Transitions
Source: J Phys Chem B. 2026 Jul 1;130(28):7250–61. doi: 10.1021/acs.jpcb.6c01531 (PMC13383716; doi:10.1021/acs.jpcb.6c01531)
Supplement: Supplementary file 1 [file jp6c01531_si_001.pdf]

Supplementary Information

The Role of Functional Groups in Substituted Benzoic Acids Used as Dopants in Liquid Crystal Mixtures on the Nematic-Isotropic Transitions

María Celina Mora[1], Joshua Brenes[1], Cristopher Camacho[1] and Erick Castellón*[1,2]

Escuela de Química, Universidad de Costa Rica, 11501-2060 Costa Rica
Centro de Investigación en Ciencia y Tecnología de Materiales, Universidad de Costa Rica, 11501-2060 Costa Rica

Contents:

S1. Differential scanning calorimetry (DSC) calculations 2

S2. Calculations with infrared spectra 4

S3. Nematic-isotropic transition temperatures and enthalpies 5

S4. Coordinates of optimized structures of 4-aminobenzoic acid complexes 6

S5. Relations between nominal molar fraction and the molar fractions of monomers and dimers 10

S1. Differential scanning calorimetry (DSC) calculations

A typical differential scanning calorimetry (DSC) thermogram of a sample undergoing a phase transition is displayed in Figure S1.1. To compute the transition enthalpy ΔH through integration, the heat flux $P(T)$ signal should be corrected by subtracting the baseline $B(T)$,

$$\Delta H = \int_{T_1}^{T_2} [P(t) - B(t)]\, dt. \qquad [S1.1]$$

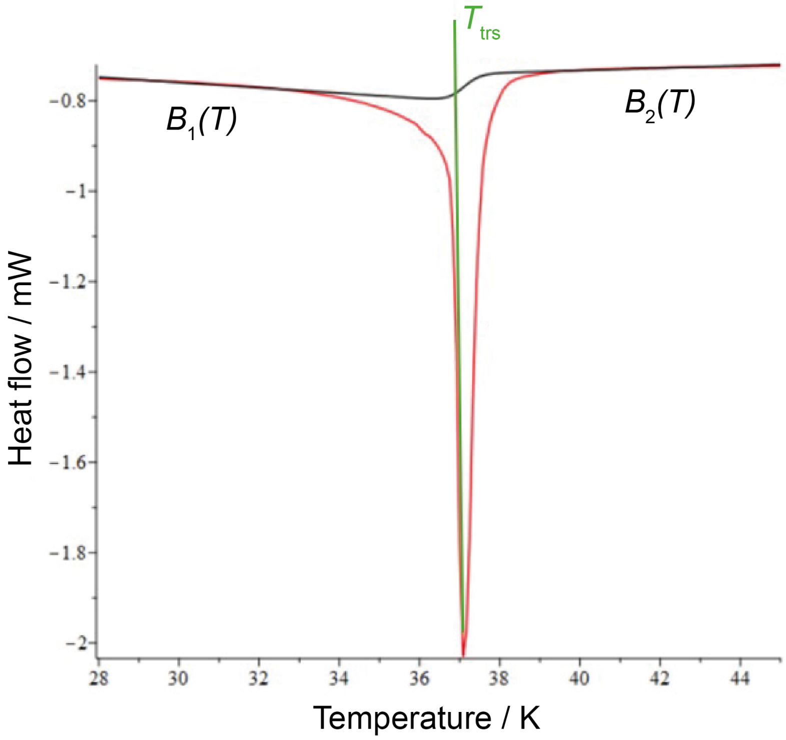

Figure S1.1. DSC thermogram showing the nematic-isotropic transition endothermic peak of a mixture of 5CB + 2-aminobenzoic acid (**1**) with nominal molar fraction $x'_A = 0.02$.

The nematic-isotropic transition enthalpies of the liquid crystal mixtures were calculated by numerical integration using the software for numerical and symbolic computation Maple. The slope of the baselines depends on the heat capacities of the samples. The heat capacity of the samples can change upon phase transitions, and thus the baseline of a DSC thermogram usually has different slopes before and after a transition. The baseline of the DSC thermograms was fitted with a composite function

$$B(T) = [1 - H(T)] \cdot B_1(T) + H(T) \cdot B_2(T), \qquad [S1.2]$$

where $B_1(T)$ and $B_2(T)$ are linear functions of temperature T, and $H(T)$ is a soft version of the Heaviside step function

$$H(T) = 1 - \frac{1}{e^{\lambda(T - T_{\mathrm{trs}})}}, \qquad [S1.3]$$

where λ is a *softening* parameter of the step function (the Heaviside function holds for $\lambda \rightarrow \infty$), T_{trs} is the transition temperature located at the minimum or maximum of the DSC thermogram depending on whether the transition is endothermic or exothermic respectively. For our calculations, the softening parameter was fixed to $\lambda = 5$.

The baselines were fitted with the equation S1.1 using the points before and after each transition peak, fixing the value of the transition temperature T_{trs}. The value of the transition temperature corresponds to the minimum or maximum of a thermogram. Once obtained the baseline function, the numerical integration of the thermograms was performed with the trapezoid method. [1]

[1] Barrante, J. R. (2004). *Applied mathematics for physical chemistry* (3rd ed.). Upper Saddle River, NJ: Pearson Prentice Hall.

S2. Calculations with infrared spectra

Figure S2.1 displays examples of infrared spectra of liquid crystal mixtures containing carboxylic acids. The signals can be deconvoluted by fitting the experimental spectra with two peak functions and a baseline.

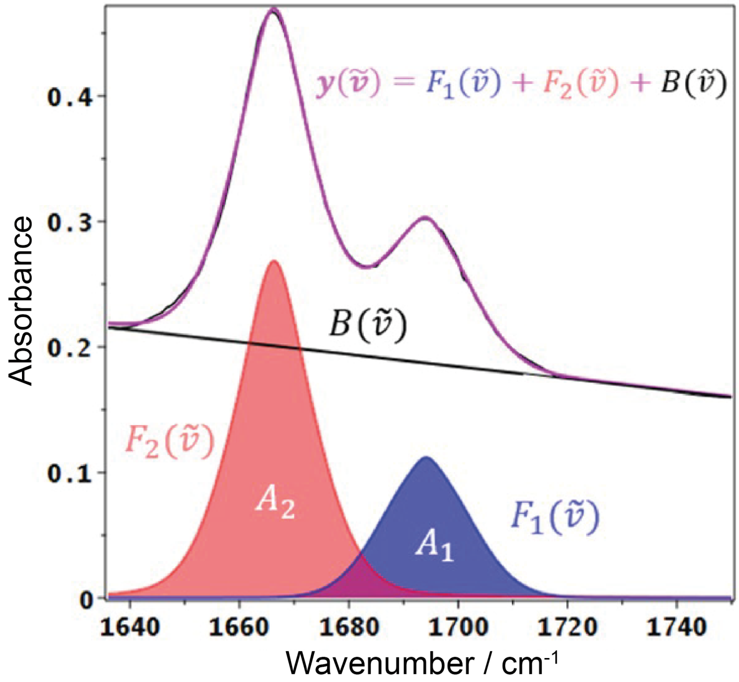

Figure S2.1. Infrared spectrum of the liquid crystal mixture 5CB + 2-aminobenzoic acid with nominal concentration $x'_{\mathrm{A}} = 0.06$ in the carbonyl stretching interval of wavenumbers $\tilde{v}$. The experimental signal is deconvoluted by the sum of a baseline $B(\tilde{v})$ and two pseudo-Voigt functions $F_1(\tilde{v})$ and $F_2(\tilde{v})$ having areas A_1 and A_2 respectively. The functions $F_1(\tilde{v})$ and $F_2(\tilde{v})$ are related to monomers and dimers respectively.

A versatile function to model spectroscopic absorption data is the pseudo-Voigt function. This function averages gaussian and lorentzian peaks with weights m_i and $1 - m_i$ respectively ($0 \leq m_i \leq 1$) for each band ($i = 1, 2$):

$$y = \sum_{i=1}^{2} A_i \left[\frac{m_i}{\sigma_{Gi}\sqrt{2\pi}} \cdot \exp\left(-\frac{(\tilde{v} - \tilde{v}_{ci})^2}{2\sigma_{Gi}^2} \right) + \frac{1 - m_i}{\pi} \cdot \frac{2\sigma_{Li}}{4(\tilde{v} - \tilde{v}_{ci})^2 + \sigma_{Li}^2} \right] \tag{S2.1}$$

the other parameters are the peak centers $\tilde{v}_{ci}$, areas A_i, gaussian widths σ_{Gi}, and lorentzian widths σ_{Li}. Subindex $i = 1$ is used to tag carbonyl bands on monomers, and subindex $i = 2$ assigned to dimeric carbonyls.

The baselines are constructed by fitting data about the extremum wavenumbers of the selected interval (1630 – 1760 cm^{-1}). The 5CB liquid crystal does not show conspicuous absorptions in the selected interval. However, a small signal due to 5CB overlaps in the spectra of the mixtures containing aminobenzoic acids. This signal is subtracted from

these spectra prior the numerical treatment. The pseudo-Voigt functions are obtained by non-linear regression using the software Maple.

S3. Nematic-isotropic transition temperatures and enthalpies

Table S3.1. Changes of the nematic-isotropic transition temperature (relative to the transition temperature of pure 5CB, $\Delta T_{\mathrm{NI}} = T_{\mathrm{NI}} - T_{\mathrm{NI}}^{*}$) and enthalpies $\Delta_{\mathrm{NI}}H$ for 5CB doped with substituted benzoic acids (**1-20**) at several concentrations. The reported enthalpies are averages computed from the results at three levels of dopant concentration. The uncertainties are the standard deviations of these measurements.

Mixture	x'_{A}	$\Delta T_{\mathrm{NI}}/\mathrm{K}$	$\Delta_{\mathrm{NI}}H$ / (kJ/mol)	Mixture	x'_{A}	$\Delta T_{\mathrm{NI}}/\mathrm{K}$	$\Delta_{\mathrm{NI}}H$ / (kJ/mol)
5CB+**1**	0.02	1.08	0.53 ± 0.01	5CB+**11**	0.02	-2.50	0.52 ± 0.02
	0.04	1.83			0.04	-3.67	
	0.06	2.67			0.06	-3.83	
5CB+**2**	0.02	1.67	0.58 ± 0.02	5CB+**12**	0.02	-1.17	0.53 ± 0.03
	0.03	1.71			0.04	-2.00	
	0.04	1.83			0.06	-2.83	
5CB+**3**	0.02	3.83	0.59 ± 0.01	5CB+**13**	0.005	0.83	0.57 ± 0.03
	0.03	6.17			0.01	0.67	
	0.04	8.33			0.02	0.50	
5CB+**4**	0.02	4.50	0.57 ± 0.01	5CB+**14**	0.02	0.17	0.50 ± 0.04
	0.04	8.83			0.04	-1.00	
	0.06	12.83			0.06	-2.00	
5CB+**5**	0.02	-1.00	0.55 ± 0.02	5CB+**15**	0.005	0.33	0.51 ± 0.03
	0.04	-2.33			0.01	0.17	
	0.06	-4.20			0.02	-0.50	
5CB+**6**	0.02	0.83	0.56 ± 0.02	5CB+**16**	0.02	0.50	0.50 ± 0.02
	0.04	0.17			0.04	1.00	
	0.06	-0.50			0.06	1.33	
5CB+**7**	0.02	0.83	0.52 ± 0.02	5CB+**17**	0.02	-2.17	0.57 ± 0.01
	0.04	1.00			0.03	-2.27	
	0.06	1.17			0.04	-2.33	
5CB+**8**	0.02	0.50	0.55 ± 0.02	5CB+**18**	0.02	1.00	0.51 ± 0.02
	0.04	1.00			0.03	1.33	
	0.06	1.50			0.04	1.50	
5CB+**9**	0.02	0.83	0.56 ± 0.02	5CB+**19**	0.02	0.30	0.52 ± 0.04
	0.04	1.17			0.03	0.50	
	0.06	1.50			0.04	0.83	
5CB+**10**	0.03	1.83	0.524 ± 0.003	5CB+**20**	0.02	0.33	0.52 ± 0.02
	0.04	2.33			0.03	0.83	
	0.06	2.00			0.04	1.00	

S4. Coordinates of optimized structures of 4-aminobenzoic acid complexes

The following structures of dimers of 4-aminobenzoic acid were optimized through quantum chemical calculations (details in Experimental methods):

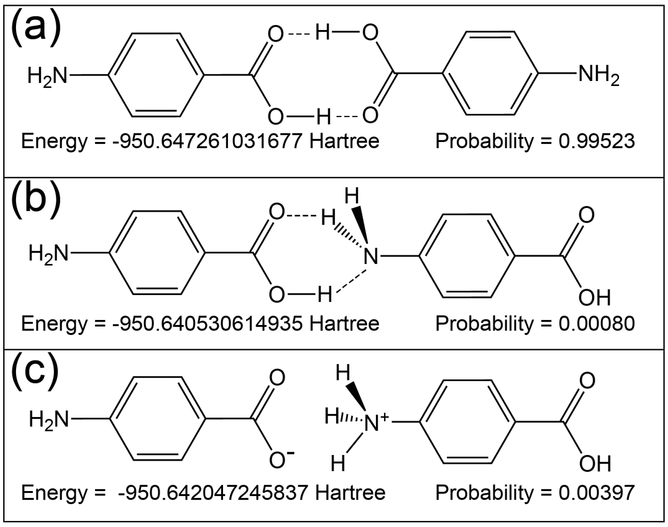

Figure S4.1. (a) Symmetric dimer with carboxylic-carboxylic hydrogen-bonding. (b) Asymmetric dimer with carboxylic-amino hydrogen-bonding. (c) Anion-cation interaction.

The following tables display the coordinates of the complexes in Figure S4.1.

Table S4.1. Coordinates of optimized structure of symmetric dimer with carboxylic-carboxylic hydrogen-bonding (Figure S4.1(a))

Atom	x / Å	y / Å	z / Å
O	1.27602727349905	-1.04904246003328	0.27830362909582
H	-0.32709643276328	-1.08297634603535	0.03802905469262
O	-1.31863631799099	-1.18732071338566	-0.06721328942844
C	-1.90421592876219	-0.01738050988095	0.12771146364113
C	-3.37436361335706	-0.06247570732342	0.11842459099491
C	-4.09221547527864	1.06027888837971	0.54232797395432
C	-5.47702328404250	1.04232893250441	0.55768241278101
C	-6.18441962940098	-0.09244070721905	0.13113180827558
N	-7.56684717803280	-0.13071200791203	0.19956458478799
H	-8.01495137853632	0.77209973750696	0.16174282090357
H	-8.00081860664995	-0.78976170544566	-0.42869093887169
C	-5.45646649393790	-1.21049896733290	-0.30346022543853
C	-4.07057880175550	-1.20072599454257	-0.30154613953044
O	-1.27322937762554	1.02821361631905	0.30906330179816
H	0.32681028604353	1.07013017498167	0.08335147274557
O	1.31903935929440	1.17939592698173	-0.01365049435534
C	1.90559031367160	0.00340433500742	0.13470724980516
C	3.37572973585907	0.05139889412246	0.12170785173696
C	4.09787474002096	-1.07790883180630	0.51959873073964
C	5.48273946834598	-1.05632123284295	0.53055043553897
C	6.18576712830287	0.08843893497668	0.12400398986961
N	7.56832661007218	0.12884644757813	0.18863724786878
H	8.01867095554083	-0.77182972546207	0.13111016493503
H	7.99900753697446	0.80182750760370	-0.42695866664664
C	5.45330381749881	1.21287661495734	-0.28577634821642
C	4.06748690835307	1.19964805124903	-0.27795075521657
H	-3.55653079109641	1.94118138243207	0.86693604348714
H	-6.02576807716127	1.91020691821626	0.90023058632538
H	-5.98954116825511	-2.09010197370524	-0.64083301035805
H	-3.52734445112502	-2.07197856037793	-0.63737400577746
H	3.56558553139566	-1.96678185521369	0.82769613117484
H	6.03501764471136	-1.92931271664402	0.85391230552035
H	5.98270018031729	2.10034488844763	-0.60788057685191
H	3.52069951587031	2.07595876389885	-0.59426939998106

Table S4.2. Coordinates of optimized structure of asymmetric dimer with carboxylic-amino hydrogen-bonding (Figure S4.1(b))

Atom	x / Å	y / Å	z / Å
C	-6.99778000327751	-1.61464948265670	0.05144142952030
C	-6.67545586174695	-1.74258025570556	-1.30425770533103
C	-7.52708793566290	-1.23517636155398	-2.27485546262781
C	-8.70526521851512	-0.57818772940259	-1.90940319905453
C	-9.59246620036141	-0.07049621204260	-2.97792609196840
O	-9.39241259165839	-0.20635063507275	-4.17042339292493
O	-10.67560980851260	0.56947718686659	-2.49552687393062
H	-11.19363081352200	0.85322481325050	-3.26567303985957
C	-9.01795062726299	-0.43062305525567	-0.55491469214540
C	-8.16764345942643	-0.94261589195435	0.41653112685659
N	-6.12032572989128	-2.11131990050403	1.03089699754602
H	-6.57291740398090	-2.25619031552950	1.92436664080593
H	-5.66606536883950	-2.96894099346577	0.74175708685217
H	-5.75785410224744	-2.24101718929749	-1.58494462268271
H	-7.28480465795308	-1.34417348737960	-3.32249779038201
H	-9.92669790832952	0.07180217199373	-0.25911102865788
H	-8.41844202085198	-0.83821516281581	1.46444956197981
O	-3.29190989897896	-1.65265683543827	-0.55597303943458
C	-0.51250055935000	-1.22010528329470	-0.17574017642755
H	-0.83582717269885	-1.60832155149215	-1.13126978404017
C	0.83654716769067	-1.03161252404590	0.08040672695836
C	-2.89456832787045	-1.17985949744991	0.49951919284061
C	-1.46943119302122	-0.92328148448523	0.79901178203120
H	1.57080105086288	-1.26752891917068	-0.67944180714342
O	-3.71843413761358	-0.86028779622683	1.50335754863945
C	1.26759666051227	-0.54152189979534	1.32172405718311
C	-1.04694037544307	-0.42989659185092	2.03712009207662
H	-4.62570130643735	-1.20962135020154	1.26302891206192
N	2.61281486775364	-0.29907619659863	1.55981991665216
C	0.30307538307157	-0.24076181258864	2.29416715564122
H	3.24051114799582	-0.85334242053391	0.99710928868751
H	-1.77489250788723	-0.20169492719770	2.80191850482675
H	2.87323046778326	-0.32247063421837	2.53408726313108
H	0.62192844567119	0.13930222511439	3.25627542231978

Table S4.3. Coordinates of optimized structure of asymmetric dimer due anion-cation interaction (Figure S4.1(c))

Atom	x / Å	y / Å	z / Å
C	-5.98050920356930	1.07737701092344	-0.71919572765227
C	-6.36826637145805	1.09902658713343	-2.06295494650355
C	-7.36376347142872	0.24632279082741	-2.51711107073314
C	-7.98987669033991	-0.63905302605270	-1.63574721063630
C	-9.05198711228399	-1.52042709121106	-2.16618811015577
O	-9.35241827409766	-1.60981488795411	-3.34192935608752
O	-9.67377646849064	-2.22896971090839	-1.20371052207633
H	-10.33668329846490	-2.77963429113721	-1.65019046557522
C	-7.60036039529738	-0.66664975188991	-0.29361414698803
C	-6.59633881603336	0.18010843819412	0.15901368058475
N	-4.92739822806381	1.89430886375456	-0.27285986835330
H	-3.50577874223412	0.91239536050345	-0.34076564478104
H	-4.98822762054413	2.11256872648719	0.71324777931680
H	-5.88829688280893	1.78801939665187	-2.74565961543809
H	-7.66751038703686	0.26400754954124	-3.55398307419693
H	-8.07662901323057	-1.34820355744558	0.39543213712316
H	-6.28558507805749	0.15332197892288	1.19430586482164
O	-2.71843377856271	0.29254051531771	-0.40970425075263
C	-0.71861914968067	-1.60518192255732	-0.37346324085997
H	-0.82164973528043	-0.97628800696413	-1.24575426201602
C	0.30129705922036	-2.54415225239920	-0.31877048609330
C	-2.72026793903000	-0.48955265640787	0.67315630095721
C	-1.61595893505709	-1.47345670210001	0.69080493150712
H	0.99069402381812	-2.64129358427277	-1.14785843186463
O	-3.55643852474651	-0.39444169865640	1.56054368118936
C	0.45509282962995	-3.37375401979411	0.80131410489151
C	-1.46976722360393	-2.30089453954174	1.80767783799610
N	1.51468550623992	-4.26520199767532	0.88319280449437
C	-0.44863146211121	-3.23612643941252	1.86547216489858
H	1.87624802340120	-4.57002014116701	-0.00745792244976
H	-2.16505921355198	-2.20398298588123	2.62980958646032
H	1.36025784772442	-5.04203493275460	1.50768875692347
H	-0.34262365143172	-3.87039235393274	2.73640349955403
H	-4.82796259703742	2.74428040175871	-0.81274668360459

S5. Relations between nominal molar fraction and the molar fractions of monomers and dimers

```
> restart
```

The nominal preparation molar fraction of dopant acid A is expressed in terms of moles of A and 5CB as:

```
> e1 := x´__A = n´__A/(n´__A + n__5CB)
```

$$e1 := x'_A = \frac{n'_A}{n'_A + n_{5CB}} \quad (1)$$

As dopant A can dimerize, the moles of acid A are distributed among monomers and dimers:

```
> e2 := n´__A = n__A + 2*n__A2
```

$$e2 := n'_A = n_A + 2\,n_{A2} \quad (2)$$

The total amount of moles of substances in the mixtures is:

```
> e3 := n__T = n__A + n__A2 + n__5CB
```

$$e3 := n_T = n_A + n_{A2} + n_{5CB} \quad (3)$$

Implying that the actual molar fraction of monomers is expressed as:

```
> e4 := x__A = n__A/n__T
```

$$e4 := x_A = \frac{n_A}{n_T} \quad (4)$$

The actual molar fraction of dimers is expressed as:

```
> e5 := x__A2 = n__A2/n__T
```

$$e5 := x_{A2} = \frac{n_{A2}}{n_T} \quad (5)$$

And the actual molar fraction of 5CB is:

```
> e6 := x__5CB = n__5CB/n__T
```

$$e6 := x_{5CB} = \frac{n_{5CB}}{n_T} \quad (6)$$

The substitution of equations e4 and e5 in equation e2 gives:

```
> e7 := subs([isolate(e4, n__A), isolate(e5, n__A2)], e2)
```

$$e7 := n'_A = x_A\,n_T + 2\,x_{A2}\,n_T \quad (7)$$

Substituting the moles of 5CB expressed in equation e6 into equation e1 yields:

```
> e8 := subs([isolate(e6, n__5CB)], e1)
```

$$e8 := x'_A = \frac{n'_A}{x_{5CB}\,n_T + n'_A} \quad (8)$$

From this latter equation e8, the nominal amount of dopant A can be isolated:

```
> e9 := isolate(e8, n´__A)
```

$$e9 := n'_A = -\frac{n_T\,x_{5CB}\,x'_A}{-1 + x'_A} \quad (9)$$

Substituting the result of equation e9 in equation e7 produces:

```
> e10 := subs(e9, e7)
```

$$e10 := -\frac{n_T x_{5CB} x'_A}{-1 + x'_A} = x_A n_T + 2\, x_{A2} n_T \tag{10}$$

From this equation, the nominal preparation molar fraction of dopant can be isolated:

```
> e11 := isolate(e10, x´__A)
```

$$e11 := x'_A = \frac{x_A + 2\, x_{A2}}{x_{5CB} + x_A + 2\, x_{A2}} \tag{11}$$

Finally, the substitution of the 5CB molar fraction in terms of the monomer and dimer molar fractions gives the expression:

```
> subs(x__5CB = 1 - x__A - x__A2, e11)
```

$$x'_A = \frac{x_A + 2\, x_{A2}}{1 + x_{A2}} \tag{12}$$
